# Strategies of functionalized GelMA-based bioinks for bone regeneration: Recent advances and future perspectives

**DOI:** 10.1016/j.bioactmat.2024.04.032

**Published:** 2024-05-09

**Authors:** Yaru Zhu, Xingge Yu, Hao Liu, Junjun Li, Mazaher Gholipourmalekabadi, Kaili Lin, Changyong Yuan, Penglai Wang

**Affiliations:** a School of Stomatology, Xuzhou Medical University, Affiliated Stomatological Hospital of Xuzhou Medical University, Xuzhou, China; b Quanzhou Women's and Children's Hospital, Quanzhou, China; c Department of Oral and Cranio-maxillofacial Science, Shanghai Ninth People's Hospital, Shanghai Jiao Tong University School of Medicine, College of Stomatology, Shanghai Jiao Tong University, National Center for Stomatology, National Clinical Research Center for Oral Diseases, Shanghai Key Laboratory of Stomatology, Research Unit of Oral and Maxillofacial Regenerative Medicine, Chinese Academy of Medical Sciences, Shanghai, China; d Cellular and Molecular Research Center, Iran University of Medical Sciences, Department of Medical Biotechnology, Faculty of Allied Medicine, Tehran, Iran

**Keywords:** GelMA, Functionalization, Bone regeneration, Stimuli-responsive, 3D bioprinting

## Abstract

Gelatin methacryloyl (GelMA) hydrogels is a widely used bioink because of its good biological properties and tunable physicochemical properties, which has been widely used in a variety of tissue engineering and tissue regeneration. However, pure GelMA is limited by the weak mechanical strength and the lack of continuous osteogenic induction environment, which is difficult to meet the needs of bone repair. Moreover, GelMA hydrogels are unable to respond to complex stimuli and therefore are unable to adapt to physiological and pathological microenvironments. This review focused on the functionalization strategies of GelMA hydrogel based bioinks for bone regeneration. The synthesis process of GelMA hydrogel was described in details, and various functional methods to meet the requirements of bone regeneration, including mechanical strength, porosity, vascularization, osteogenic differentiation, and immunoregulation for patient specific repair, etc. In addition, the response strategies of smart GelMA-based bioinks to external physical stimulation and internal pathological microenvironment stimulation, as well as the functionalization strategies of GelMA hydrogel to achieve both disease treatment and bone regeneration in the presence of various common diseases (such as inflammation, infection, tumor) are also briefly reviewed. Finally, we emphasized the current challenges and possible exploration directions of GelMA-based bioinks for bone regeneration.

## Highlights

• The functionalization strategy of GelMA based bioink for bone tissue engineering was summarized.
• The response strategies of GelMA to external physical stimulation and internal pathological microenvironment.
• Major challenges and exploration directions of GelMA for bone regeneration.

## Graphical abstract

This paper summarized the strategies for functionalization of GelMA-based bioinks, including property enhancement, stimulus response and adaptation to specific pathological microenvironments.

**Figure:**
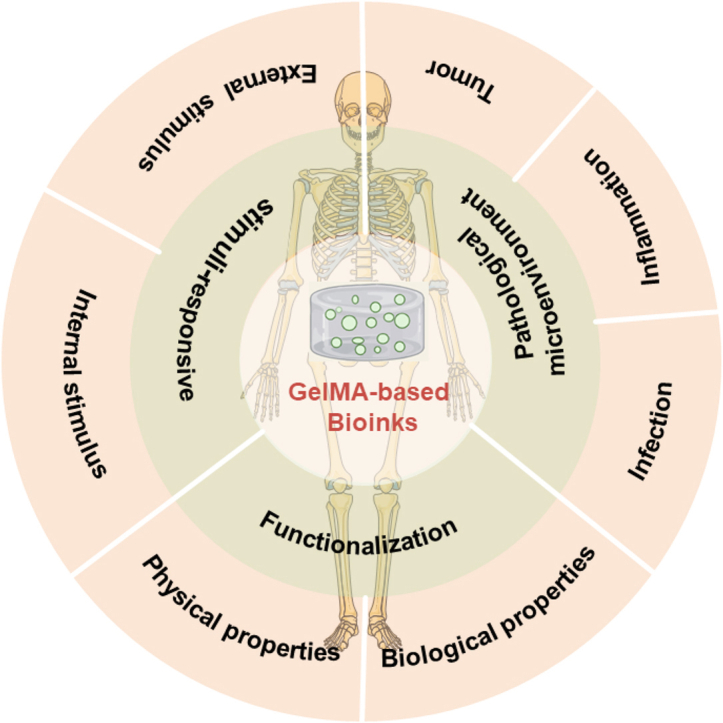

## 1 Introduction

Bone is an important tissue and organ of the human body, which serves as the scaffold for the body, and undertakes the functions of support, load-bearing, hematopoiesis and metabolism. However, bone defects caused by tumors, trauma, inflammation and congenital diseases are one of the major threats to human health. At present, the main method of bone defect repair is bone transplantation, including autograft and allograft [1]. Although bone tissue is the second most common type of graft tissue [2], the traditional bone graft repair methods still have many problems such as infection, immune rejection, sacrifice of healthy tissue, insufficient supply and so on [3]. Artificial bone scaffold transplantation can effectively solve the lack of donor source. The ideal artificial bone scaffold should not only fill the bone defect area, bear the mechanical load function of the site, but also promote the bone regeneration of the defect area and restore the physiological function of the site. Traditional methods of artificial bone manufacturing (solution casting, pneumatic hole molding, etc.) suffer from a series of problems such as low structural precision, long processing cycles, difficulty in matching patients, and lack of physiologically active function [4]. In recent years, bone tissue engineering has gradually become a new research hotspot, which opens up new prospects for solving the bone regeneration problem by using various biomaterials to prepare biological scaffolds with bone regeneration function.

With the development of three-dimensional (3D) printing technology in the 1980s, bone scaffolds with ideal structural accuracy can be customized through 3D printing technology [5]. Although 3D printing technology has made breakthrough progress, it has not yet achieved expectations. Printing scaffolds still have limitations, such as the lack of bioactivity and biomimetic properties. For this reason, “3D bioprinting”, which incorporates biologically active substances, has been developed rapidly in recent years. 3D bioprinting not only enables precise control of the shape, structure, and spatial location of the target object, but also, and most importantly, is biologically active and can mimic tissues or organs to achieve bionic functions [6]. According to different prototyping principles and printing materials, 3D bioprinting is primarily based on three core methods: extrusion-based bioprinting, droplet-based bioprinting, and photocuring-based bioprinting [7]. In extrusion-based bioprinting, a bioprinter extrudes bioinks to form continuous filaments for constructing scaffolds. Droplet-based bioprinting generates discrete droplets that stack to form scaffold structures. Photocuring-based bioprinting uses photocurable materials to solidify and stack layer by layer, conducting scaffolds. Extrusion-based bioprinting is widely used due to its ease of operation, relatively low cost, and the wide range of available biomaterials [8].

In recent years, the development of 3D bioprinting technology in the field of bone tissue engineering has opened up new prospects for solving the problem of bone regeneration [9]. Currently, one of the major challenges facing bone tissue engineering bioprinting is the lack of ideal bioinks that can match the biomechanical property and bioactivity of bone tissue [10]. Bioinks are a specialized type of biomaterial primarily used in 3D bioprinting technology. They are mainly composed of biodegradable polymers, bioactive materials, and growth factors, aimed at providing structural support during the printing process and offering a growth environment and signals to promote cell growth, differentiation, and tissue regeneration. Bioinks should have good biocompatibility, and flexible extrudability and injectability, which are important basic conditions for bioprinting. There are a variety of biomaterials that can be used as bioinks, including collagen, chitosan, alginate, cellulose, polymer and so on. Hydrogels are widely used in bone tissue engineering due to their good biocompatibility, degradability and water retention and a series of properties similar to extracellular matrix [11]. Among them, GelMA is a representative hydrogel preparation, which not only possesses flexible injectability but also exhibits excellent printability, and is currently a more mature bioink, thus showing great potential for application in bone tissue engineering. GelMA hydrogels is a modified gelatin obtained by the reaction of gelatin with methacrylic anhydride (MA), which is suitable as a bioink because of the characteristics of photo-crosslinking, excellent biocompatibility, low immune response and printability. More importantly, GelMA hydrogels have many properties conducive to osteogenesis. Such as being highly similar to natural extracellular matrix,which can simulate conditions for *in vitro* cell culture; with good biocompatibility, which can enhance cell viability; and retention of arginine glycine aspartate (RGD) sequences and matrix metalloproteinase (MMP) sequences [12], which support certain cell behaviors (e.g., adhesion, proliferation, differentiation, etc.). These properties make GelMA an ideal platform for studying cellular osteogenic differentiation, by providing attachment sites and signaling cues to guide cell growth and osteogenesis. Previous studies have demonstrated that GelMA can effectively promote osteogenic differentiation of MSCs [13], BMSCs [14], and hMSCs [15].

It is well known that the osteogenic capacity of pure GelMA is relatively limited due to its weak mechanical properties, lack of a continuous osteo-inductive microenvironment and immunomodulatory ability. Therefore, in order to make GelMA-based hydrogels better used in bone tissue engineering, it is necessary to functionalize and improve the properties of GelMA-based hydrogels, such as physical properties (including mechanical strength, self-healing, and viscoelasticity) and biological properties (including vascularization ability, osteo-inductive ability, immunomodulatory ability, and neural vascularization regeneration ability). “3D bioprinting technology”, as a transformative technology, is currently the most concerned research hotspot in the field of bone tissue engineering, which is expected to create bone biological scaffold that can accurately mimic natural bone tissue structure. Therefore, it is very important to pay attention to and improve the bioprinting performance of GelMA-based bioinks.

Moreover, the survival microenvironment continues to transmit various stimulus signals to the hydrogel, which is another important factor affecting the function of GelMA hydrogel. Therefore, this paper also briefly reviewed the response strategies of GelMA hydrogel to external physical stimulation and internal pathological microenvironment stimulation, as well as the functional strategies of GelMA hydrogel to achieve both disease treatment and bone regeneration in the presence of various common diseases (such as inflammation, infection, and tumor).

In conclusion, this paper mainly reviewed the research progress of functionalized bone tissue regeneration strategies of GelMA-based bioinks, and proposed the challenges and future prospects of the applications of GelMA functionalization strategies in bone regeneration.

## 2 Synthesis and structure of GelMA

**Fig. 1:**
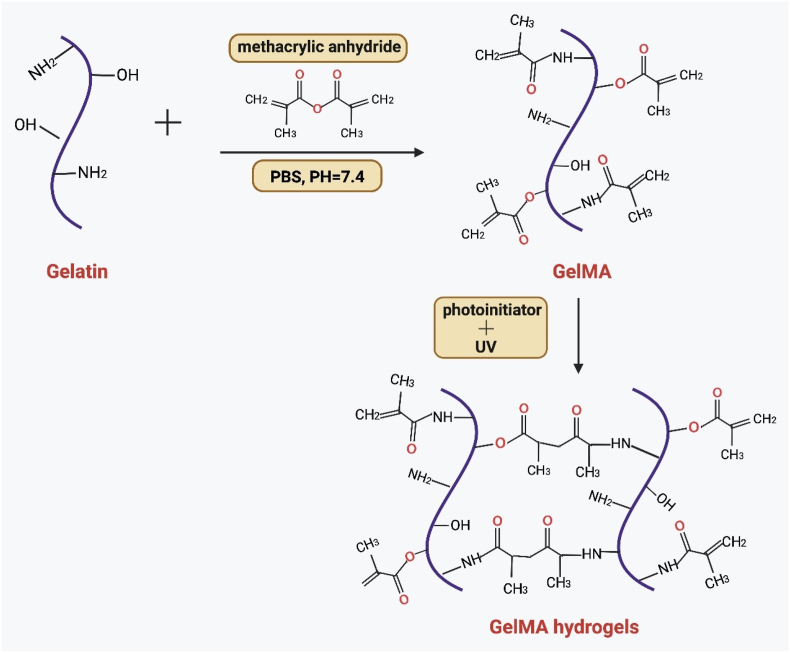
Scheme for preparation of photo-crosslinked GelMA hydrogel.

At present, the most widely used GelMA synthesis method is the traditional method proposed by Van Den Bulcke et al. [16] in 2000. In traditional preparation methods, gelatin was completely dissolved in phosphate buffered saline (PBS, pH = 7.4) at 50 °C, methacrylic anhydride (MA) was slowly added to the gelatin solution to undergo a substitution reaction (Fig. 1). The reaction was terminated by dilution with PBS, then the diluted solution was dialyzed to remove unreacted MA and other toxic by-products. Finally, the dialysate was freeze-dried for storage [17]. This methacryloyl modified gelatin allows the GelMA molecule covalently crosslink in the presence of light and photoinitiator, to obtain a stable morphology. Common photoinitiators of GelMA hydrogels include 2-hydroxy-1-[4-(2-hydroxyethoxy) phenyl]-2-methyl-1-propanone (Irgacure 2959) and lithium phenyl-2,4,6-trimethylbenzoylphosphinate (LAP) [18]. In comparison to I2959, LAP has a better water solubility and enhanced kinetics, which increased stability and print fidelity [19].

In addition to traditional synthesis methods, several synthesis strategies optimized around traditional methods have been reported in recent years, which increase the degree of MA substitution by replacing PBS with dimethyl sulfoxide (DMSO), carbonate-bicarbonate buffer (CB) or reverse osmosis (RO) [20] filtered water as the solvent, or reduce cell damage by ultraviolet (UV) by optimizing the photoinitiation system. The differences of these methods compared with traditional preparation methods are summarized in Table 1.

**Table 1:** Summary of the optimization strategy of GelMA's synthesis methods.

Synthesis method of GelMA	Advantage	Disadvantage	Ref.
Traditional preparation method	Simple preparation method Low cost Non-toxic by-products	Long term exposure to ultraviolet light damages cells	Van Den Bulcke, Bogdanov, De Rooze, Schacht, Cornelissen and Berghmans [16]
DMSO substituted for PBS as solvent	Increase the degree of MA substitution	Low production rate	Martineau, Peng and Shek [21]
CB substituted for PBS as solvent	Increase the degree of MA substitution	The pH value of the solution needs to be monitored and regulated	Lee, Shirahama, Cho and Tan [22]
RO substituted for PBS as solvent	Fast photocrosslinkability and high stiffness	Lower degradation rates	Hitendra Kumar, Kabilan Sakthivel and Mohamed G. A [20]
Eosin Y photoinitiated system	Crosslinking with visible light Better cytocompatibility	High concentrations of EY, TEOA, VC are toxic	Sharifi, Sharifi, Akbari and Chodosh [23]

**Abbreviations:** DMSO: dimethyl sulfoxide; CB: carbonate-bicarbonate buffer; EY: Eosin Y (photosensitizer); TEOA: triethanolamine.

## 3 Functionalization strategies of GelMA-based bioinks for bone regeneration

**Fig. 2:**
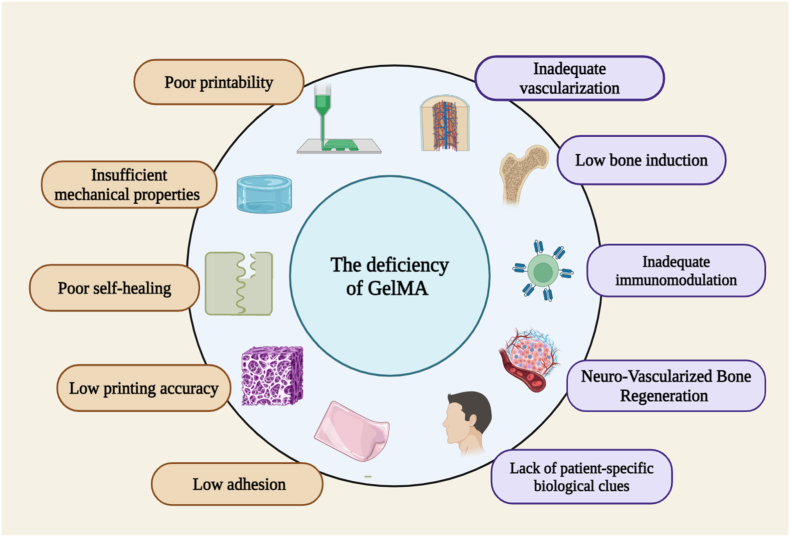
Summary of deficiencies with GelMA hydrogel in bone tissue engineering application.

Although GelMA-based bioink has good printability and biocompatibility, there are still some remarkably restrictive factors in the application of pure GelMA hydrogel in bone regeneration. There are many shortcomings in the physical properties of GelMA hydrogels, such as limited printable range, poor mechanical strength and printing accuracy, uncontrollable porosity, low viscoelasticity, self-healing and adhesion. In terms of biological properties, it is faced with some deficiencies as well, such as low bone induction, insufficient vascularization ability, lack of immunomodulatory modifiers factors and patient specific biological clues and so on (Fig. 2). Therefore, there is a need to tailor the GelMA hydrogel synthesis process or add various biomaterials for functionalized modification of GelMA hydrogels to meet the requirements of bone tissue applications. In this section, the functionalization strategies to improve the physical and biological properties of GelMA-based bioinks for bone regeneration are summarized.

### 3.1 Improving the physical properties of GelMA for bone regeneration

**Fig. 3:**
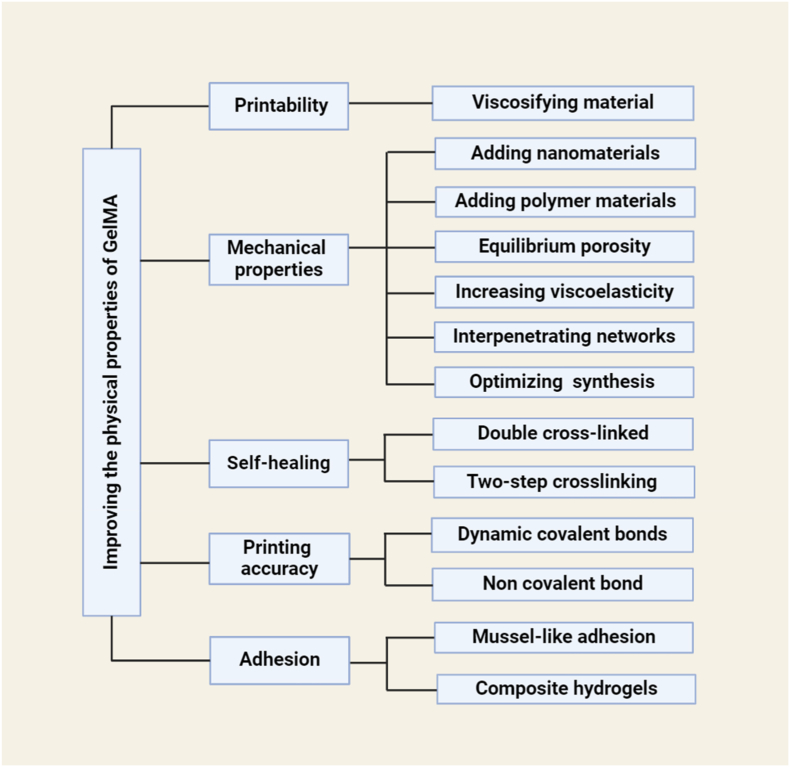
Schematic of the main optimization strategies for GelMA physical properties.

The physical properties of GelMA hydrogel are important factors affecting the printing process as well as the structure and function of the scaffold. Excellent printability is a prerequisite for GelMA hydrogel to be used as a bioinks. While the mechanical strength, porosity, and high precision of GelMA hydrogel determine whether the printed structure can provide sufficient mechanical support and create a complex biomimetic microenvironment conducive to material exchange for the loading cells. In addition, viscoelasticity affects the direction of cell differentiation, while self-healing and adhesion are important factors to ensure the stable existence of hydrogels in the repair environment. In fact, in addition to the stable swelling and degradation properties of GelMA hydrogel, its printability, mechanical strength, porosity, viscoelasticity, self-healing, accuracy and adhesion are not enough to meet the needs of bone regeneration, and it needs to be further functionalized to improve its physical properties. This section summarizes the main optimization strategies for improving various physical properties of GelMA, including printability, mechanical properties, self-healing, print accuracy and adhesion (Fig. 3).

#### 3.1.1 Improving the printability of GelMA

Printability is the prerequisite of materials as bioinks. To satisfy printing eligibility, GelMA hydrogel should have the main following features: (1) Shear thinning behavior allows hydrogel to reduce viscosity during extrusion, so as to avoid cell damage caused by high shear stress. (2) high fidelity, which enable hydrogels restores initial viscosity after printing to maintain the shape of printing structure and avoid collapse deformation. (3) cross-linking ability, giving hydrogel the ability to form a permanent network. (4) biocompatibility.

It is well known that GelMA hydrogel has good biocompatibility and crosslinking characteristics, but it still has problems in printability such as insufficient shear thinning behavior, low viscosity at mild temperature and poor fidelity. More importantly, GelMA is a temperature sensitive material, and the control of temperature is closely related to the printability of GelMA hydrogels. The ideal temperature range for printing GelMA hydrogels with different concentrations is very limited, for example, the theoretical printing temperature of 10 % w/v GelMA is at 18 °C–19 °C. While once the optimum printing temperature is exceeded, GelMA bioink is difficult to form regular filaments, resulting in poor printability [24]. Janmaleki et al. [25] investigated the effect of different printing temperatures on the printing structure of GelMA hydrogel, and the results showed that the temperature range for GelMA hydrogels to print were very limited.

Moreover, they proposed that GelMA had good printing fidelity at low temperatures, but low cellular compatibility. Therefore, how to expand the bioprinting window of GelMA hydrogel under the premise of considering both print fidelity and cell compatibility is a problem to be solved urgently.

Rastin et al. [26] successfully improved the printing applicability of GelMA by preparing photo-crosslinked MC/GelMA hydrogels using methylcellulose (MC). MC is a water-soluble polymer with good printing suitability and can undergo sol-gel transformation at 37 °C. However, MC lacks crosslinking ability, which is difficult to be maintained for a long time. In MC/GelMA composite bioinks, MC acts as viscosifier to provide GelMA with printing suitability, while GelMA's cross-linking ability helps MC maintain biostability. The interaction between MC and GelMA hydrogel improved the printing suitability of GelMA hydrogel. In another strategy, GelMA hydrogel is added with gelatin and the viscosity of GelMA bioink is adjusted by changing the mixture ratio of gelatin. This method enables the low concentration GelMA/gelatin mixed bioinks to obtain good printing performance, which brings benefit for cell loading [27].

In addition to improving printing suitability, the fidelity of printing structure should also be taken into account. Xanthan gum is a natural extracellular polysaccharide with high shear dilution behavior and high viscosity. Garcia-Cruz et al. [28] prepared composite bioinks through a chemical bonding network between modified xanthan gum (XGMA) of methacrylate and GelMA hydrogel. The high viscosity and shear dilution properties of XGMA improve the printing stability of GelMA hydrogel, and maintain high shape fidelity and cellular activity after printing. Nanoclay is a kind of nano-sized silicate, which has unique thixotropic properties, that is, the viscosity will decrease when nanoclay is subjected to stress changes, and the viscosity will return to its original state after the stress is stabilized. Therefore, it has good shear thinning behavior, which can improve the printing suitability of bioinks and maintain good structural fidelity [29]. Gao et al. [30] defined the printable window of GelMA-nanoclay mixed bioinks through systematic experiments and demonstrated that the addition of nanoclay balanced the printing performance and biocompatibility while ensuring high fidelity. In spite of this, nanoclay-based GelMA bioinks were also limited by the strict printing temperature. For this reason, Dong et al. [31] further developed GelMA/Laponite composite bioink by adding a synthetic magnesium silicate clay. They screened the optimum mixing ratio of 15 % GelMA and 8 % Laponite, which can achieve good printing performance under mild conditions. While improving printing applicability, damage to cellular activity by strict printing temperature should be avoided. And the properties of temperature-sensitive materials are expected to resolve this conflict. Wang et al. [32] prepared mixed bioinks using elastin, *o*-nitrobenzyl (NB)-grafted hyaluronic acid, and GelMA, which not only improved the printing performance, but also significantly promoted cellular activities such as cell spreading, phenotype maintenance, and tissue regeneration.

The above research shows that adding materials with viscosity-increasing or high shear-thinning behavior is an effective strategy to improve the printability of GelMA. The key point is to ensure cell activity, structural fidelity and mild printing conditions while improving the printability. The application of temperature-sensitive materials is expected to solve this challenge and shows excellent potential in improving the printability of GelMA.

#### 3.1.2 Improving the mechanical properties of GelMA

The GelMA hydrogel itself has some degree of mechanical tunability, but the range of tunability is relatively limited. The microstructure of the scaffold can be varied by the concentration, degree of substitution，porosity and viscoelasticity of the GelMA hydrogel, resulting a greater degree of mechanical adjustability can be obtained, by affecting the microstructure of the scaffold. However, as a gelatin-based hydrogel, the poor mechanical properties of GelMA often fails to meet the needs of bioprinting for bone tissue engineering and bone regeneration. As a gelatin-based hydrogel, the poor mechanical properties of GelMA often fails to meet the needs of bioprinting for bone tissue engineering and bone regeneration. Many strategies have been developed to improve the mechanical properties of GelMA to meet the biological functions.

**Fig. 4:**
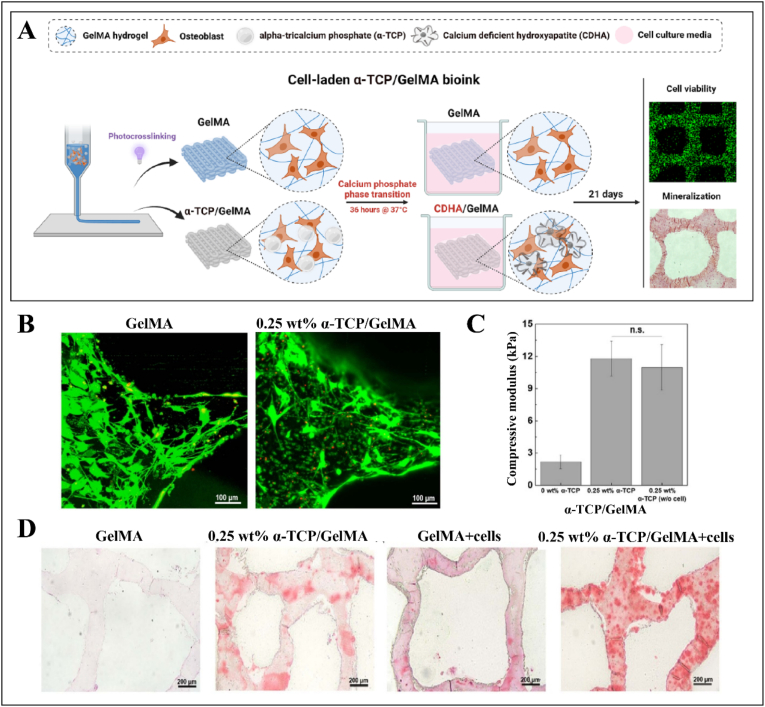
A) Schematic image of the osteogenesis-enhancing α-TCP/GelMA bioink and the CDHA/GelMA produced via the calcium. B) Live/dead cell assay results for the fabricated scaffolds of GelMA and 0.25 wt% α-TCP/GelMA. C) A comparison of the compressive modulus values of the scaffolds. D) *In vitro* osteogenic cellular activities were assessed using Alizarin Red S stained. [reprinted with permission from Kim et al. [39] copyright (2024) Biofabrication].

**Fig. 5:**
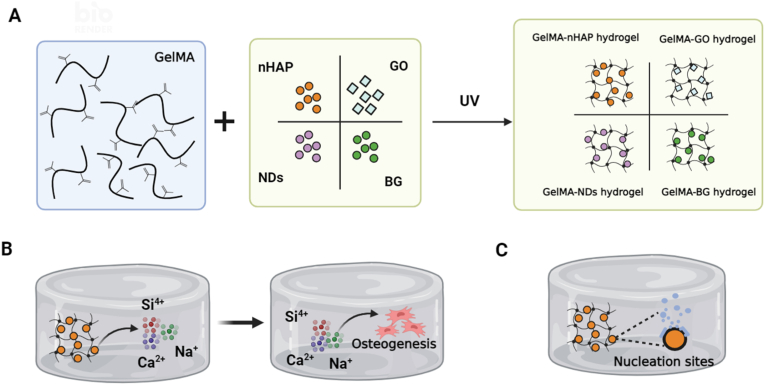
A) Nanoparticles (nHAP, GO, BG, NDs, BG) interact physically or chemically with the GelMA hydrogel network to enhance the mechanical strength of GelMA hydrogel. B) Some nanoparticles release ions, which can promote the osteogenic differentiation of stem cells. C) Nanoparticles in GelMA hydrogel provide nucleation sites for bone matrix calcification.

Nanomaterials are widely used to enhance the mechanical characteristics of hydrogels since they can stride across multiple polymer chains to effectively disperse stress across the network, thus increasing the mechanical strength of hydrogels [33]. The specific applications of these nanomaterials in GelMA hydrogel are summarized in Table 2. Among them, one-dimensional nanomaterials, such as carbon nanotubes and nanowires, mainly improve mechanical properties by forming covalent bonds with GelMA [34]. Recently, Yu et al. [35] demonstrated that strontium-doped xonotlite [Ca_6_Si_6_O_17_(OH)_2_] (Sr-CSH) nanofibers can enhance the mechanical properties of GelMA and increase the mechanical strength of GelMA hydrogel by more than two times. More importantly, in their follow-up studies [36], it was proved that GelMA doped with Sr-CSH has good printability and is a bioinks with great potential. While two-dimensional nanomaterials like graphene oxide (GOs) can also significantly enhanced the mechanical properties of GelMA hydrogels due to the strong adherence between GOs and the acrylic groups on the GelMA chains [37,38]. Kim et al. [39] used 0.25 wt% tricalcium α-phosphate (α-TCP) and 5 % GelMA to develop a novel bioinks for bioprinting bone scaffolds. α-TCP was converted to the calcium-deficient hydroxyapatite (CDHA) precipitate, by a phase transition from calcium superphosphate, which not only resulted in an 8-fold increase in the mechanical properties of the GelMA hydrogel, but also led to a significant increase in the level of osteogenic differentiation of the encapsulated cells (Fig. 4). In addition, nanoparticles such as nano hydroxyapatite (nHAp) [40], bioactive glass (BG) [41] and nanodiamond (NDs) [42], etc, can interact with GelMA hydrogel network in physical or chemical way to improve the mechanical properties and keep stability of the hydrogels (Fig. 5A). In conclusion, in addition to enhancing the mechanical properties of GelMA hydrogels, more importantly, some of these nanomaterials can release functional ions that help stem cells differentiate into osteogenic cells (Fig. 5B), while the presence of nanomaterials can also provide a nuclear site for mineralization of the bone matrix, thus promoting bone formation (Fig. 5C).

**Table 2:** Summary of the different strategies to improve mechanical properties for GelMA hydrogels.

Biomaterials	Parameter	Advantages	Ref.
GO	0.07 (w/v) % GO, 5 (w/v) % GelMA	Improve mechanical properties (increased by ∼2-fold) Enhance electroactivity Enhance the toughness	Xavier Mendes et al. [61]
HAp	20 mg/ml HAp, 5 (w/v) % GelMA	Improve mechanical properties (increased by ∼1-fold) Improves cell proliferation	Suvarnapathaki et al. [62]
BG	10 (w/v) % BG, 10 (w/v) % GelMA	Improve mechanical properties (increased by ∼4.4-fold) Reduce degradation rate Promote cell attachment	Zheng et al. [37]
NDs	0.2 (w/v) % NDs, 7 (w/v) % GelMA	Improve mechanical properties (increased by ∼2-fold) Reduce degradation rate	Pacelli et al. [63]
CNTs	0.5 mg/ml CNTs, 5 (w/v) % GelMA	Improve mechanical properties (increased by ∼3-fold) Stronger cell adhesion	Shin et al. [31]
α-TCP	0.25 wt % α-TCP 5 (w/v) % GelMA	Improve mechanical properties (increased by ∼8-fold) Stable swelling performance	Kim et al. [39]
Sr-CSH	4 (w/v) % Sr-CSH, 10 (w/v) % GelMA	Improve mechanical properties (increased by ∼2-fold) Enhance stability	Yu et al. [35]
PCL	50000 g/mol PCL 15 (w/v) % GelMA	Improve mechanical properties (increased by ∼100-fold)	Pacelli et al. [43]
HAMA	2 (w/v) % HAMA, 10 (w/v) % GelMA	Improve mechanical properties (increased by ∼2.5-fold) High cell viability	Liu et al. [64]
CS	2 (w/v) % CS, 10 (w/v) % GelMA	Improve mechanical properties (increased by ∼28-fold) Excellent biocompatibility	Fan et al. [46]
Alg	2 (w/v) % Alg, 10 (w/v) % GelMA	Improve mechanical properties (increased by ∼3-fold) Improve toughness and elasticity	Suo et al. [47]
Microwave assistance	1000W/4%MA hydrogels	Improve mechanical properties (increased by ∼2-fold) Degradation resistance	Mamaghani et al. [65]
PACG	35 (w/v) % PACG, 7 (w/v) % GelMA	Improve mechanical properties (increased by ∼3-fold)	Ying et al. [66]

Polymer materials are often used as auxiliary materials because of their excellent mechanical properties. For example, polycaprolactone (PCL) can provide mechanical strength for GelMA hydrogel to meet the needs of bone tissue by interval printing [43]. Alcala-Orozco et al. [44] modified PCL with Mg(OH)_2_ nanoparticles for the first time. This kind of nanocomposite bioinks (Mg-PCL-GelMA) could further generate a mechanical reinforcement 30 %–400 %. Although thermoplastic materials can provide excellent mechanical strength for GelMA hydrogels, the printing methods of thermoplastic materials are more complex, and printing at high temperature may affect the cell activity of the hydrogel. Therefore, in order to reduce the negative impact on cell activity, Fan et al. [45] developed a mixed biological ink containing GelMA, hyaluronic acid and cellulose nanocrystals. In which, cellulose nanocrystals enhance mechanical properties by applying electrostatic and attraction between two kinds of hydrogels. This hybrid printing method provided good mechanical support for the printing structure on the basis of ensuring the good biological activity of cells.

Another way to enhance the mechanical characteristics of GelMA hydrogels is to construct hydrogels with interpenetrating networks (IPN). On a molecular level, GelMA with a high cytocompatibility is combined with another tough hydrogel. Compared with single GelMA hydrogel, IPN hydrogel exhibited excellent stability and mechanical strength. There have been many attempts on IPN hydrogels, such as GelMA-chitosan (CS) [46], GelMA-alginate (Alg) [47] and other IPN hydrogels show good mechanical reinforcement and structural stability. partial dynamic cross-linking. In a recent study, Mamaghani et al. [48] added GO nanoparticles to GelMA/PEGDA hydrogels. The dense polymer network formed by PEGDA/GelMA makes it obtain ultra-high strength (24-fold increase in mechanical properties), and the addition of GO can not only help to enhance the mechanical strength, but also improve the electrical conductivity.

Other than the using of auxiliary materials and interpenetrating network, mechanical properties can also be enhanced by optimizing the synthesis method of GelMA hydrogels. For example, selecting the methacrylic acid process of gelatin assisted by microwave energy (Mw) can increase the methacrylic acid degree of GelMA, so as to obtain higher mechanical properties [49]. Gao et al. [50] successfully developed a unique biodegradable and supramolecular hydrogen bonding enhanced GelMA hydrogels by copolymerization of poly (N-acryloyl 2-glycine) (PACG) and GelMA, the dihydrogen bond of PACG could considerably strengthen and stiffen the intrinsically weak GelMA hydrogel.

In fact, researchers are more interested in achieving precise tuning of the mechanical strength of printed structures to meet the requirements of different target tissues than in simply improving the mechanical properties of the printed structures. Based on this, Wang et al. [51] proposed a new method to first mix GelMA and hyaluronic acid methacrylate (HAMA) for bioprinting, and then use hyaluronidase (Hase) to digest HAMA in the structure, thus achieving precise modulation of a wide range of mechanical properties (above 100 kPa down to about 1 kPa). More importantly, there is no damage to the fidelity and biological activity of the printed structures after enzymatic digestion. This method promises to bring the printed structures closer to the complexity of real organs, rather than just printed structures with homogeneous mechanical properties. In summary, they demonstrated that the method can achieve widely tunable and precisely controllable mechanical properties to meet the mechanical requirements of organs such as the heart, lungs, and brain.

The porosity and pore size of GelMA hydrogel scaffolds impact the viability, proliferation and differentiation of the loaded cells. Studies have shown that GelMA hydrogel with high porosity and large pore size (>100 μm) are more conducive to promoting osteogenesis because the larger surface area can lead to more ion exchange and bone induced protein adsorption [52]. It is well known that the porosity of GelMA hydrogel largely depends on its own synthetic conditions. For instance, the GelMA samples cured with Irgacure 2959 have a higher pore size compared to those cured with LAP [53]. Celikkin et al. [54] also reported that the degree of substitution and concentration of GelMA hydrogel were negatively correlated with pore size. Besides, GelMA hydrogels synthesized at lower temperatures, longer UV exposure or for longer freezing time [55] resulted in smaller pore sizes. In conclusion, GelMA hydrogel concentration, degree of substitution and cross-linking conditions are all factors that affect the porosity, but these factors are less adjustable. To better adjust the pore size of GelMA hydrogel, a relatively mature method is to prepare an aqueous two-phase emulsion bioink including GelMA and poly (ethylene oxide) (PEO), which obtained porous structure through removing the PEO after photo-crosslinked [56]. By adjusting the volume ratio of PEO to GelMA, the pore size of GelMA hydrogel is highly tunable, which can meet different pore size and porosity requirements. Although the porous GelMA structure has the disadvantage of poor mechanical properties, but the mechanical strength can be enhanced by adding other nanomaterials. In general, GelMA-PEO biphasic emulsion bioink is still an ideal pore adjustment strategy. However, it is still a great challenge to maintain the enough mechanical strength of GelMA with high porosity and large pore size.

Viscoelasticity is the inherent property of GelMA hydrogel affecting cell reaction. In printed structures, the initial adhesion of cells to the extracellular matrix will be subject to the resistance of the matrix (elastic response), but this resistance will weaken over time (viscous response) [57]. Extracellular matrix (ECM) can provide mechanical signals to internal cells through viscoelasticity to influence the direction of cell migration and differentiation. Interestingly, GelMA hydrogel viscoelasticity is not a one-way relationship but a cell-matrix interaction. Martinez-Garcia et al. [58] suggested that the viscoelasticity of GelMA hydrogel with a concentration of 5 % was more conducive to the proliferation and survival of adipose-derived stromal cells (ASCs). In addition, with the passage of time, the existence and behavior of ASCs increased the stress relaxation of GelMA hydrogel and reduced the viscoelasticity. A previous study showed that the addition of human platelet (hPL) solution to GelMA hydrogel can effectively enhance GelMA viscoelasticity, thereby promoting the migration and proliferation of fat cells. More importantly, as viscoelasticity increased, more cells in the GelMA network established cell-to-cell contact [59]. Additionally, Choi and his colleagues prepared a composite scaffold that was more suitable for bone tissue ingrowth using GelMA and silanated silica, and they demonstrated that the composite scaffold significantly increased viscoelasticity, mechanical strength, cell proliferation and osteogenic differentiation [60]. In conclusion, an appropriate increase in viscoelasticity of GelMA hydrogel can promote cell adhesion, proliferation and the establishment of more cell-cell contact, which are conducive to osteogenic differentiation of cells.

It is well known that the good extrudability and injectability of GelMA is the basis for bioprinting. Although GelMA in some of the above studies was used in a direct injection manner, the problems explored and solved are still important guides and references for GelMA bioprinting technology. We believe that the ultimate goal of these related studies is to create ideal bioprinted bone scaffolds in combination with bioprinting technology. In summary, more and more functional strategies have been employed to improve the mechanical properties of GelMA hydrogels, but some of them may have some shortcomings like complex printing methods, damage of cell activity and so on. Therefore, in the specific application of bone regeneration, it is necessary to select a more appropriate functionalization strategy combined with the characteristics of the target bone tissue. For example, when repairing the defects of large load-bearing bones (such as limb bones), selecting thermoplastic materials for mixed printing can meet the needs of high mechanical properties. On the contrary, when repairing bone defects with lower hardness and higher degree of vascularization (such as alveolar bone), nanomaterials with the function of promoting osteogenesis and vascularization seem to be the better choice.

#### 3.1.3 Improving the self-healing of GelMA

Self-healing means that hydrogels can spontaneously restore their original structure and function by forming dynamic connections after being damaged by physical or biological factors [67]. In the environment of bone regeneration, hydrogels often face various challenges, including damage from physical (e.g., stress) and biological factors (e.g., bacteria, enzymes, etc.), while the self-healing function of hydrogels can prevent loss of structure and function in the process of bone regeneration [68]. In addition, traumatic bone defects are often accompanied by soft tissue injury and bleeding, and the self-healing function of hydrogel can also play a role in promoting soft tissue healing and hemostasis in such cases.

At present, the self-healing mechanism of hydrogels basically relies on the same principle, that is, through the mediation of non-covalent bonds or dynamic covalent bonds, to the fractured matrix of hydrogels is rejoined [67]. Among them, non-covalent bonds are weak connections from intermolecular, which can be reconstructed rapidly after dissociation, mainly including hydrogen bonds, ionic bonds, and host-guest interactions, etc [69]. However, GelMA hydrogels do not have ideal self-healing properties [70]. GelMA photo-crosslinking is an irreversible process that will turn off its weakly self-healing ability after photo-crosslinking and stabilize the hydrogel for long-term cell culture [71]. GelMA itself has no non-covalent or dynamic bonds, and it is difficult to reorganize covalent bonds in the event of fracture, and the accumulation of tiny cracks will eventually lead to GelMA fracture [72]. Therefore, current approaches to improve the self-healing function of GelMA hydrogels rely primarily on binding to hydrogels with available dynamic covalent bonds.

Wang et al. [72] prepared three-armed supramolecular (HGSM) hydrogels via host-guest interactions between ethyl acrylate modified β-cyclodextrin and arylation of tetramethylene glycol-modified adamantane. On this basis, the host-guest supramolecular hydrogels (HGGelMA) were formed by covalent cross-linking of HGM with GelMA. In HGGelMA, the covalent cross-linking maintains the overall shape, while the reversible weak interaction between the non-covalent host-guest not only enhances the mechanical properties, but also can be re-established during fracturing to heal the hydrogel. Similarly, Ren et al. [73] recently used β- Cyclodextrin grafted alginate (ALG- β CD) and N-adamantyl acrylamide (Ad AAm) to form a host-guest unit, and then covalently cross-linked with GelMA to obtain composite hydrogels. In addition to the good self-healing function brought by the host-guest interaction, the composite hydrogel also shows high tensile deformation ability and 3D printing ability, and is considered as a potential tissue engineering material.

**Fig. 6:**
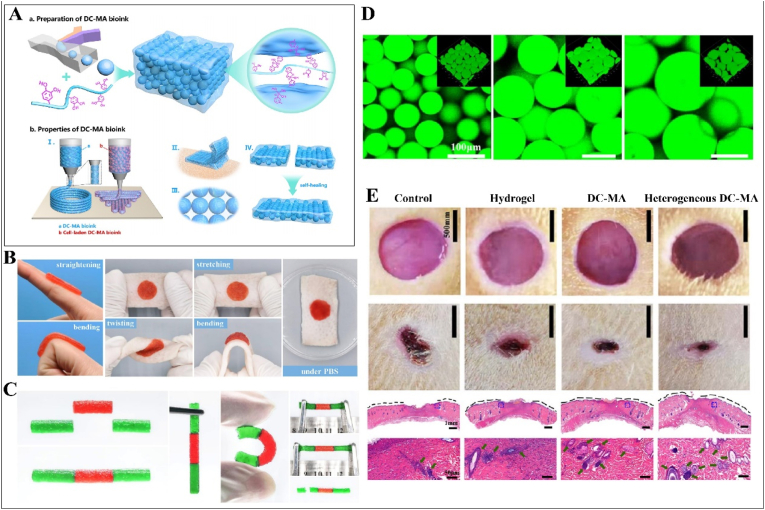
A) Schematic diagram of DC-MA bioink preparation and properties. B) Tissue-adhesion of DC-MA bioink. C) Self-healing of DC-MA rods. D) Microporosity of DC-MA bioink. E) Wound healing using a heterogeneous DC-MA layer. Gross wound images on day 0 and day 14, and HE staining images. [reprinted with permission from Feng et al. [75]; copyright (2022) ACS Appl Mater Interfaces].

Compared to normal covalent bonds, dynamic covalent bonds have the ability to reconnect in the absence of physical stimuli. Thus, dynamic covalent bonds have both the stability of covalent bonds and the reversibility of non-covalent bonds, which can give hydrogels the ability to “self-healing”. Common dynamic covalent bonds include imine bonds, hydrazone bonds, borate ester bonds, etc. Wang et al. [74] prepared dual network hydrogels by blending amino and aldehyde-modified hyaluronic acid (HA) and GelMA co-blended and applied this DN hydrogel for 3D printing. This strategy utilized HA cross-linked by dynamic hydrazone bonds to enable the scaffold to obtain self-healing function and improve printability, while the static network of GelMA hydrogel provided good stability and compatibility of the structure. In addition, Feng et al. [75] assembled bioink (DC-MA) by cross-linking methacrylate and phenylboronic acid modified hyaluronic acid with GelMA via droplet microfluidics and adding a dynamic cross-linking agent (dopamine-modified hyaluronic acid, HA-DA). The dynamic covalent bonding enabled the printed structures to obtain good self-healing properties, adhesion and microporosity, and the printed structures also showed excellent wound healing *in vivo* experiments (Fig. 6). In a recent study, Li et al. [76] developed a self-healing hydrogel system loaded with angiogenic peptide (QK) and osteogenic peptide (KP). The dynamic imine bonding between gelatin methacrylate and oxidized dextran enabled the composite hydrogel to have self-healing ability, and more importantly, the expected release profile of KP and QK immobilized on the composite hydrogel was obtained through the Schiff base reaction of imine bonding, which significantly improved the osteogenic differentiation of BMSCs and the angiogenic ability of HUVECs. Moreover, KP and QK in self-healing hydrogels showed significant synergistic effects in rat cranial new bone formation.

At present, the main strategy to confer self-healing function to GelMA lies in the copolymerization of GelMA with hydrogels with self-healing properties to form double network hydrogels. However, there are still some problems to be solved, such as the local dynamic crosslinking only shows reversibility under mild conditions, and the degree of self-healing is limited.

#### 3.1.4 Improving the printing accuracy of GelMA

**Fig. 7:**
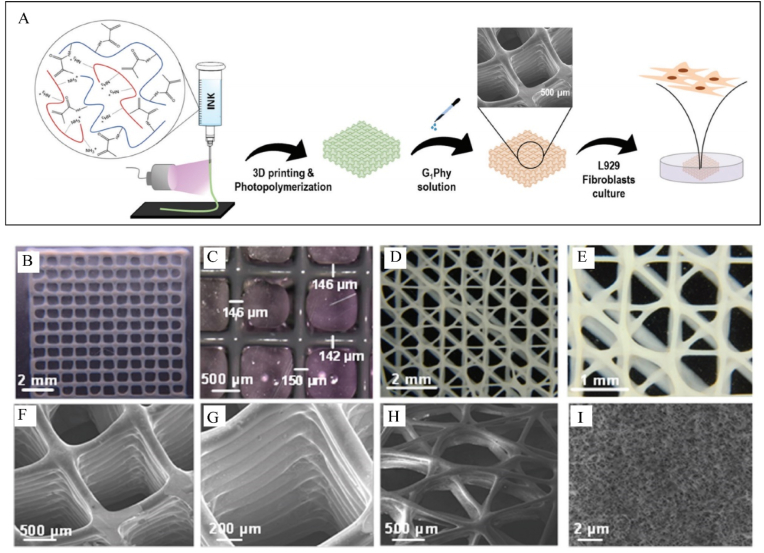
A) 3D printing approach applied for GelMA/chitosan 3D polymeric scaffolds. Light microscopy pictures of a 4-layer GelMA/chitosan scaffold at low B) and D) and higher C) and E) resolution (150 μm needle). F) and G) Images of cryo-SEM micrographs of 3D printed scaffolds (28 layers with quadrangular geometry). H) Images of cryo-SEM micrographs of 3D printed scaffolds (4 layers with angular geometry). I) The microstructure of the printed hydrogel. [reprinted with permission from Mora-Boza et al. [77]; copyright (2018) Biomaterials Science].

The prerequisite for building complex bionic structures is excellent printing accuracy, but as one of the most attractive advantages of 3D printing, the printing accuracy is often reduced due to insufficient performance of the printing process or bioinks. Extrusion-based bioprinting stands as the most extensively applied technique in contemporary biofabrication. Its primary advantage lies in its capacity to print a diverse array of biocompatible materials, encompassing cell aggregates, cell-laden hydrogels, microcarriers, decellularized matrix constituents, and more, spanning a viscosity spectrum from 30 mPa/s to 6 × 10^7^ mPa/s for biological substrates [7]. Nonetheless, relative to alternative bioprinting modalities, extrusion-based methods exhibit diminished printing precision. Another important issue to note is that high-viscosity bioinks, such as higher concentrations of GelMA, offer better formability and structural support for printed constructs, thereby enhancing printing accuracy. On the other hand, low-viscosity materials provide a suitable environment for maintaining cell viability and functionality. Therefore, researchers are exploring feasible solutions to achieve a balance between cell activity and printing accuracy. Mora-Boza et al. [77] used GelMA and chitosan as bioinks and used a novel cross-linking agent, glycerol phytate (G_1_Phy), to prepare double cross-linked scaffolds. They first polymerized the gelatin using UV crosslinking and then treated it with G_1_Phy for ionic crosslinking, and this method allowed excellent printing accuracy (resolution ≈150 μm) of the printed structures. Moreover, the uniform ionic cross-linking between the phosphate group of G_1_Phy and the amine group of GelMA and chitosan facilitates the control of the water absorption of the printed structures, which is very important for the long-term maintenance of the printing accuracy (Fig. 7).

The focus of another study is on improving the printing methods. Jin et al. [78] developed a new two-step crosslinking method for the construction of small-diameter bionic vascular networks. First, a planar structure and a concave structure were printed separately, then the planar structure was cross-linked by UV light for a short time to obtain a certain support strength, and finally, the planar structure was covered on top of the concave structure and cross-linked by light again for 100 s to combine the two structures. Through this method they have successfully bioprinted complex bionic vascular networks with high precision, providing new ideas for building small diameter blood vessels or other more complex organs.

The above research shows that improving the printing strategy of GelMA is an effective way to improve the printing accuracy of complex structures. However, while improving the printing method to obtain high printing accuracy, cell vitality should also be taken into account, which is the key to bioprinting technology.

However, the achievable precision of extrusion-based bioprinting is limited, typically in the range of 100 μm. Moreover, the inevitable shear forces during extrusion may impact cell viability, especially evident when printing bioinks with high cell densities. In contrast, photocuring-based bioprinting, such as Digital Light Processing (DLP), operates without nozzles, thus avoiding mechanical damage to cells. DLP is a surface projection-based method known for its higher printing resolution. Additionally, uncured liquid bioinks can provide excellent support for printed structures, preventing hydrogel collapse or deformation during the printing process. Wang et al. [51] developed a GelMA/HAMA composite bioink and fabricate high-precision structures by DLP. They further adapted this approach for different tissue types by selectively enzymatically digesting HAMA to achieve varying levels of tissue hardness. Hence, photocuring-based bioprinting technology is anticipated to play an increasingly significant role in cell bioprinting and has the potential to replace extrusion-based bioprinting as the predominant bio 3D printing technology in the future.

#### 3.1.5 Improving the adhesion of GelMA

The adhesion of hydrogel is an important factor that affects the effectiveness of tissue repair. When the adhesion ability is poor, it is difficult for the hydrogel to adhere to the target bone tissue and it is easy to fall off, which will seriously reduce the efficacy of bone repair [79]. The adhesion property is not only a prerequisite to ensure that GelMA hydrogel can play a long-term effective repair effect, but more importantly, when a fracture occurs, blood vessels, soft tissues and periosteum will inevitably be damaged [80]. At this time, the excellent adhesion of GelMA will also perform the function of hemostasis and promote soft tissue healing. Therefore, it is necessary to improve the adhesion of GelMA.

The mussel-like adhesion principle is one of the most commonly used strategies to enhance hydrogel adhesion, and many studies have been conducted based on it. For example, Liu et al. [81] introduced tannic acid (TA) into GelMA to prepare composite hydrogels for the first time. The catechol component in the TA structure made GelMA obtain excellent adhesion performance, and showed the best repair effect *in vivo* experiments of skin and gastric trauma. Similarly, Liu et al. [82] reported a strategy to enhance GelMA adhesion by grafting dopamine (DA) onto the GelMA macromolecular backbone, and the catechol moiety in DA conferred excellent adhesion (9.1 kPa) as well as tensile strength (2.4 MPa) to GelMA through covalent linkage. *In vivo* experiments, the biocompatible GelMA-DA hydrogel was able to tightly adhere to the skin defects of mice and accelerate wound closure.

To further impart hemostatic properties to highly adherent GelMA hydrogels, Rajabi et al. [83] prepared composite hydrogels using thiolated gelatin (Gel-SH), polydopamine-functionalized LAP (PD-LAP), and GelMA, and their results showed that the addition of PD-LAP nanoparticles not only improved the tissue adhesion of Gel-SH/GelMA hydrogels strength but also significantly reduced the clotting time (1.5 ± 0.5 min). Chang et al. [84] prepared a composite hydrogel composed of GelMA and phenyl isothiocyanate modified gelatin (Gel-Phe). The supramolecular interaction between GelMA and Gel-Phe makes the composite hydrogel firmly adhere to the bleeding site and play a hemostatic function after UV cross-linking.

In order to further combine the function of bone regeneration, Yang et al. [85] prepared biomimetic artificial periosteum by photo-crosslinking GelMA, l-arginine-based unsaturated poly (ester amide) (Arg-UPEA) and methacrylate hydroxyapatite nanoparticles (nHAMA). The combination of these three materials coordinates the superior mechanical properties and adhesion properties, so that the hydrogel can stably adhere to the fracture or bone defect, and can withstand a certain amount of pressure. More importantly, l-arginine and Ca^2+^ slowly released by Arg-UPEA and nHAMA can promote bone blood supply recovery and bone remodelin.

Despite some encouraging results regarding highly adherent GelMA hydrogels, there are still some difficult issues to be addressed. For example, highly adherent GelMA hydrogels can stick to the target bone tissue concurrently bonding to the normal surrounding soft tissue, causing unwanted adhesions. Therefore, it is hoped that in the future, GelMA with selective adhesion, or even “smart adhesion” GelMA that can achieve different adhesion abilities according to the different healing stages of different bone tissues, can be developed.

### 3.2 Improving the biological properties of GelMA for bone regeneration

**Fig. 8:**
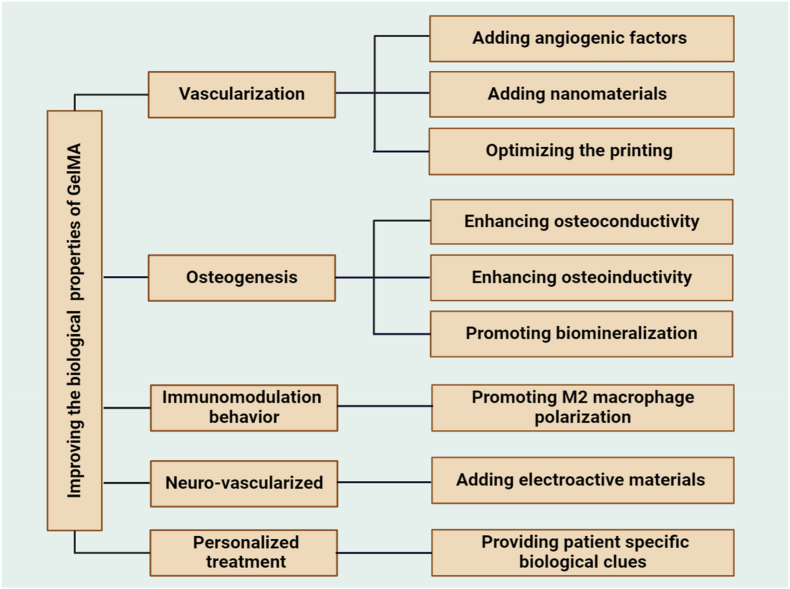
Schematic of the main optimization strategies for GelMA biological properties.

Good biocompatibility, non-toxicity and low immunogenicity are the excellent characteristics of GelMA as bioink. However, as a bone tissue-specific bioink, GelMA hydrogel lacked good sustained bone induction and rapid and effective vascularization, so it still needs further improvement. In addition, with the in-depth exploration of the biological characteristics and bone regeneration mechanism of GelMA hydrogel, through functionalized GelMA hydrogel regulates immune response to promote bone regeneration, and the development of GelMA based bioink with patient-specific biological activity clues have gradually become a research hotspot. Below, we will briefly summarize the functionalization strategies of GelMA hydrogel in terms of biological characteristics from the above four aspects. This section summarizes the main optimization strategies for improving the biological properties of GelMA, including vascularization, osteogenesis, immune behavior regulation, neuro-vascularization, and personalized treatment strategies (Fig. 8).

#### 3.2.1 Improving the vascularization of GelMA

**Fig. 9:**
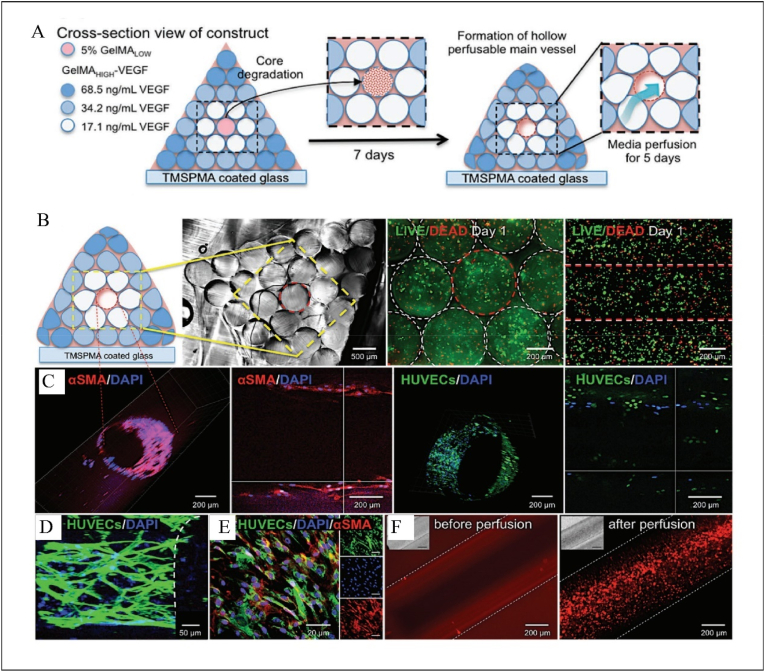
A) Schematic diagram of bioprinting vascularized bone structure. B) Encapsulated cells stained with Live/Dead are shown in cross and top views. C) The bioprinted structure has a HUVEC-lined vessel-like lumen. Encapsulated endothelial cells were lined the vascular walls (green fluorescence) and hMSC cells were differentiated into pericytes (red fluorescence). D) Formation and lining of endothelial cells inside the central channel. E) Immunostaining of endothelial cells andα-SMA-expressing hMSCs in the lumen's interior. F) 3D hydrogel images of before and after microbeads perfusion via the core hollow lumen. [reprinted with permission from Byambaaet al. [90]; copyright (2017) Advanced healthcare materials].

Natural bone is a highly vascularized tissue, and angiogenesis plays an important role in bone regeneration [86]. Therefore, the printed structures need to have rapid and effective internal vascularization capacity to avoid cell death due to hypoxia and nutrient deficiency [87,88]. GelMA hydrogel has been proven to assist the development of a vascular network based on human progenitor cells in previous investigations. What's more, the higher methacrylation degree of GelMA will leads to lower microvascular density and smaller lumen [89]. Angiogenesis stimulating substances such as vascular endothelial growth factor (VEGF) are frequently added to GelMA hydrogels in order to enhance printing structure angiogenesis. Byambaa et al. [90] used GelMA hydrogels containing different concentrations of VEGF to print the inside of the structure, and GelMA hydrogels loaded with hMSCs and silicate nanoplatelets to print the outer layer to simulate vascular-rich bone tissue constructs (Fig. 9). The results showed that the inherent gradient of VEGF in engineered constructs could enhance microcapillaries formation, while GelMA modified with silicate nanoplatelets will induce osteogenic differentiation. In addition, the addition of nanoclay into GelMA hydrogel could enhance the retention of VEGF and showed stronger angiogenesis [91].

**Fig. 10:**
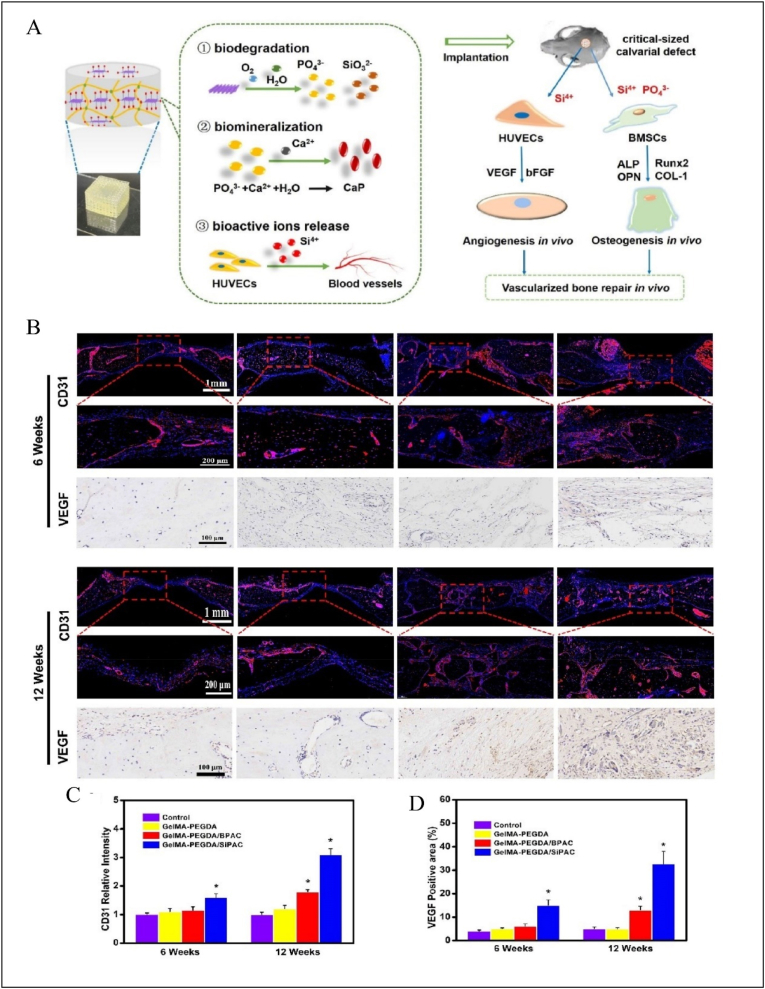
A) Angiogenesis and bone growth were urged by the release of bioactive ions from SiP nanosheets that degrade in the GelMA-PEGDA/SiPAC hybrid hydrogels. B) Immunofluorescence and immunohistochemistry detection of CD31 and VEGF expression. C) and D) Quantitative analysis of CD31 and VEGF expression level. Data represent means ± SD (n = 4). *p < 0.05 indicates significant difference compared with the control (GelMA-PEGDA) group. [reprinted with permission from Xu et al. [92]; copyright (2021) Advanced healthcare materials].

Besides angiogenic factors, some novel nanomaterials may also provide clues of biological activity for vascular bone regeneration. Recently, Xu et al. [92] prepared biohybrid hydrogels using phosphorus silicide (SiP) nanosheets and GelMA, in which continuously released silicon ions and phosphorus ions act as promoters of angiogenesis and bone formation, respectively (Fig. 10). They proposed that GelMA hydrogels combined with SiP nanosheets have good angiogenesis and osteogenesis induction.

In addition, vascularization can also be enhanced by optimizing the printing strategy of GelMA. The angiogenesis and osteogenesis bioinks based on GelMA hydrogel were prepared respectively, and then the indirect co-culture of endothelial cells and bone cells was achieved by coaxial printing of bone-like structures. The ability of the non-contact co-cultured cells could make the printing structure have higher angiogenic and osteogenic activities [93].

In order to achieve ideal angiogenesis in printed structures, in addition to enhancing the preservation of vascular growth factors, the focus is to explore printing strategies with vascular biomimetic structures to create a more conducive microenvironment for angiogenesis. However, at present, it is still difficult to print complex bionic vascular structure stably and conveniently based on GelMA system, which needs to be further explored.

#### 3.2.2 Improving the osteogenesis of GelMA

Bone regeneration is a complex process involving the recruitment, proliferation and differentiation of related cells and the formation and remodeling of bone matrix [94,95]. Pure GelMA hydrogel is difficult to provide clues for cells to promote osteogenic differentiation and mineralized matrix formation. In order to overcome these problems, GelMA hydrogel needs to be functionally modified via physical, chemical and biological approaches to better simulate osteogenic differentiation of stem cells. Improving osteogenic performance typically involves enhancing the osteoconductivity, osteoinductivity, and biomineralization of reinforced GelMA [11].

**Fig. 11:**
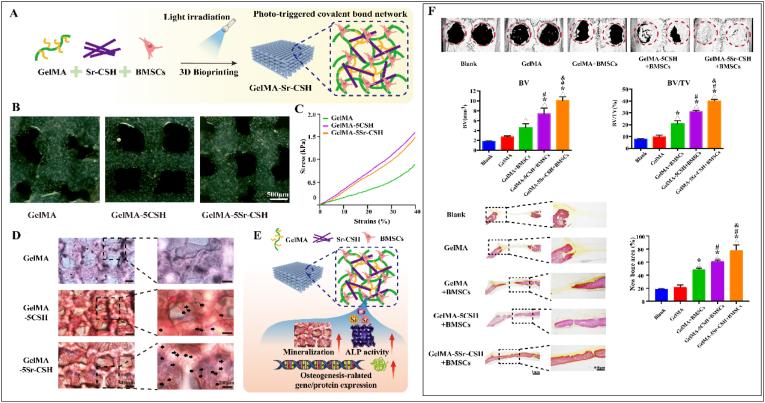
A) Schematic of GelMA-Sr-CSH bioink loaded with BMSCs for 3D bioprinting. B) Digital images of the 3D bioprinted lattice-like constructs. C) The stress-strain curve. D) The ARS staining of calcium nodules in 3D bioprinted scaffolds after 14 days of culture. E) Schematic diagram of the effects of GelMA/Sr-CSH 3D bioprinted scaffolds on osteogenesis of BMSCs. F) Micro-CT image for calvarial defects. Quantitative analysis of bone volume (BV) and bone volume fraction (BV/TV). Representative VG staining images of the resin sections. Quantitative analysis of the new bone. Δ*P* < 0.05 compared with Blank. *p < 0.05 compared with GelMA. #*P* < 0.05 compared with GelMA + BMSCs. &*P* < 0.05 compared with GelMA-5CSH + BMSCs. [reprinted with permission from Yu et al. [36]; copyright (2024) Composites Part B-Engineering].

Firstly, osteoconductivity typically refers to the ability of implanted biomaterial surfaces to support the formation of new bone tissue, primarily influenced by bone repair conditions and cell responses to the biomaterial [96]. To enhance the osteoconductivity of GelMA and improve its osteogenic activity, adding inorganic materials such as calcium phosphate ceramics and bioactive glasses is a common functionalization strategy. Among them, HAp is the most commonly used material, which has a significant positive impact on the formation and remodeling of bone matrix [97]. Literature studies suggests that HAp doped with divalent, trivalent or tetravalent ions, such as strontium ions [98,99], lithium ions [100], magnesium and calcium ions [101], etc. Can better simulate the composition of natural bone [102]. Leu Alexa et al. [103] reported a cerium ion doped composite bioink based on GelMA and HAp. They demonstrated that cerium ion modification of HAp could further improve the performance and osteogenic differentiation ability of the printed scaffold. Tavares et al. [104] used functionalized mesoporous silica nanoparticles to modify GelMA hydrogels. Mesoporous silica components could induce the formation of bone-like Hap, and the slowly released functional inorganic ions can guide the osteogenic differentiation of stem cells even when there is no osteogenic inducer present. Recently, Yu et al. [36] utilized 10 % GelMA/5 % strontium-doped calcium stibnite nanowires (Sr-CSH) as a bioink to encapsulate bone marrow mesenchymal stem cells (BMSCs) for the construction of bionic bone tissues via bioprinting. The incorporation of Sr-CSH improved the printing precision and mechanical strength of the GelMA hydrogel, and significantly promoted the osteogenic differentiation of the BMSCs in both *in vivo* and *in vitro*. More importantly, Sr-CSH induced macrophage M2 polarization, thereby modulating the inflammatory microenvironment and further promoting bone formation (Fig. 11). Nanoclay can not only improve the retention of bioactive factors, but also its degradation products have bone inductivity. Laponite-GelMA composite bioinks have been shown to load cells for stable printing and promote osteogenic differentiation in the absence of osteogenic factors [91]. More recently, Man et al. [105] used Laponite-GelMA as bioink to load epigenetic enhanced osteoblast derived extracellular vehicles (EVs). In which, EVs promoted the osteogenic differentiation of hBMSCs by increasing the acetylation level of H3K9 histone, while the stability and retention of Laponite can enhance the in-situ retention of vesicles and further promote mineralization in GelMA-LAP hydrogels. In summary, GelMA hydrogel based on nanoclay not only promotes osteogenic differentiation, but also possesses carrier function.

Secondly, osteoinductivity typically refers to the ability of a biomaterial to induce multipotent mesenchymal cells to differentiate into osteoblasts, ultimately leading to new bone formation. The addition of some bioactive molecules can trigger the biochemical reaction of cells and induce the osteogenic differentiation of stem cells. Bone morphogenetic protein-2 (BMP-2) is a common growth factor in bone tissue engineering, which can induce osteogenic differentiation of stem cells. Compared with the exogenous BMP-2 released from the medium, the BMP-2 encapsulated in GelMA hydrogel was more sustained and the local delivery, which more conducive to promoting the osteogenic differentiation of hASCs [106]. However, due to the degradation of protease, BMP-2 has a short half-life *in vivo*. In order to ensure the long-term delivery and presentation of BMP-2 in GelMA hydrogels, a more stable BMP-2 mimetic peptide can be selected instead. The short amino acid chain of BMP-2 mimetic peptide makes its structure more stable and can effectively combine with GelMA hydrogels [107]. Zhou et al. [108] maintained the bioactivity and controlled release of BMP-2 and bFGF (vascular growth factor) by means of mineral coating microparticle (MEM) on GelMA hydrogel. Their results demonstrated that the “burst” release of bFGF can promote the expression of intracellular nitric oxide (angiogenesis function index of endothelial cells) and the up regulation of angiogenesis related genes (CD31, ang, VEGF-A). Meanwhile, bFGF and BMP-2 jointly regulate the activity of osteoblast related cells, which eventually promote the coupling of angiogenesis and osteogenesis. In addition, some studies have also tried to use proteins as clues to biological activity, such as 14-3-3 *ε* protein loaded GelMA hydrogel, which have been proved to improve osteogenic differentiation of stem cells by stimulating cell adhesion and proliferation. This study provided a new idea for the application of GelMA hydrogels in bone tissue regeneration [109].

Achieve success of bone regeneration not only need osteogenesis differentiation of stem cells, cell load matrix of biomineralization is also very important. In a recent study, Subbiah et al. [110] used calcium carbonate-calcium citrate core-shell microparticles to achieve biomineralization of 3D printing GelMA hydrogels. They proved that calcium and citric ions released from microparticles could increase the apatite structure in collagen structure, thereby promoting the in-situ mineralization of GelMA hydrogels. At the same time, citrate and minerals in the mineralized microenvironment are also biological clues to induce osteogenic differentiation. In most studies, GelMA hydrogel has been successfully applied to the repair of non-load-bearing bone defects, but little has been done about segmental bone defect repair. Li et al. [111] through the segmental bone defect model in rats, it is proved that the bio-GelMA loaded with BMSC can not only strengthen the mechanical strength of bone defects, but also produce the largest and stronger new bone, which can effectively promote the repair of segmental bone defects.

In conclusion, the functional GelMA-based bioink has shown significant potential in the repair of various bone defects, including segmental bone defects.

#### 3.2.3 Improving the immunomodulation behavior of GelMA

Bone formation and remodeling are affected by the inflammatory condition of the local microenvironment, so it is very related to the host immune response produced by the scaffolds [112,113]. As we know, GelMA hydrogel is a hydrolysate of collagen, which has good biocompatibility and immunogenicity, and does not cause severe immune response. Based on this premise, many studies have attempted to promote bone regeneration by modulating immune responses through functionalized GelMA hydrogel.

Previous studies have shown that macrophages are the main regulator that mediates the immune responses of biomaterials and regulates osteogenesis. Macrophages of different phenotypes play different roles in immune response, M1 macrophages promote inflammation, while M2 macrophages are anti-inflammatory and promote tissue healing and remodeling [114]. In the immune response, a transient low level of the proinflammatory factor TNF-α (M1, M2 secretion) promotes matrix mineralization, so temporary inflammation is essential to enhance osteogenesis [115]. However, the long-term existence of pro-inflammatory immune cells will hinder the regeneration of bone tissue. Therefore, it is very important to promote the transition of macrophages from M1 phenotype to M2 phenotype, so as to complete the transformation from temporary inflammation to tissue regeneration microenvironment, which is very important for bone regeneration. Jiang et al. [116] prepared functional GelMA hydrogels containing platelet plasma (PRP), which promoted the differentiation of monocytes into macrophages M2 by PRP, and achieved the transformation of macrophages from M1 phenotype to M2 phenotype. They demonstrated that 3D-printed PRP-GelMA scaffolds enhanced the osteogenic capacity of BMSCs through immune regulation.

**Fig. 12:**
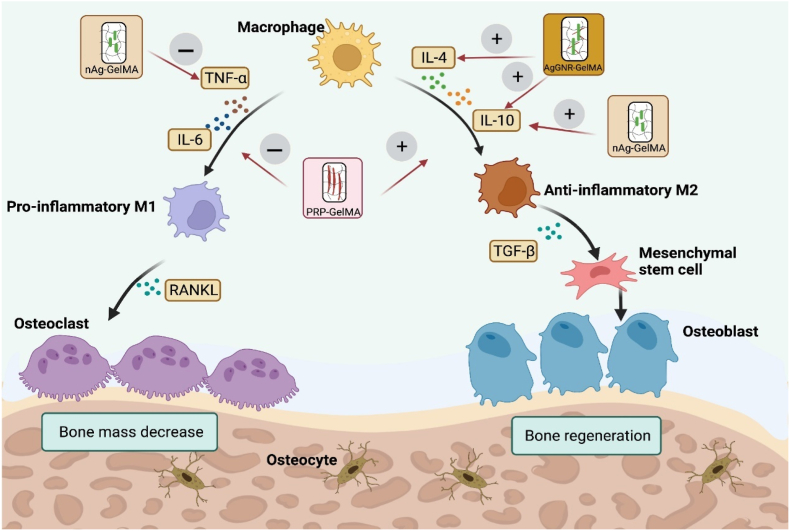
Schematic diagram of macrophages influencing bone regeneration by modulating immune microenvironment.

In a previous study, nano-silver (nAg) in nAg/GelMA composite hydrogels played an immunomodulatory role by inhibiting TNF-α secretion and promoting IL-10 secretion, thereby promoting the osteogenic differentiation of periodontal membrane stem cells [117]. In order to further create an immune microenvironment conducive to osteogenesis, Wang et al. [118] demonstrated the successful loading of Interleukin-4 (IL-4) onto silver-coated gold nanorods and their incorporation into bioinks composed of GelMA and dextran, which IL-4 and MSCs could synergistically promote the differentiation of macrophages into M2 phenotype. This study highlighted the excellent biocompatibility, printability, and immunomodulatory capabilities of the developed bioink system. In conclusion, this composite bioink possessed good antibacterial and anti-inflammatory properties through immune regulation. The promoted transition of the macrophages from M1 phenotype to M2 phenotype brought benefit to osteogenic differentiation of MSCs. In summary, functional GelMA hydrogel regulates the immune response mainly by creating conditions conducive to the polarization of M2 macrophages, so as to achieve a microenvironment conducive for bone regeneration (Fig. 12).

Macrophage polarization is regulated by multiple signals, with the most extensively studied signals including lipopolysaccharide (LPS), interferon-γ (IFN-γ), and interleukin-4 (IL-4) [119]. LPS primarily promotes M1 polarization of macrophages by activating the nuclear factor kappa-light-chain-enhancer of activated B cells (NF-κB) signaling pathways, leading to the production of pro-inflammatory cytokines. In the classical NF-κB signaling pathway activation, inhibitor of NF-κB (IκB) is phosphorylated by inhibitor of NF-κB (IKK), leading to its degradation. As a result, NF-κB is released from the cytoplasmic NF-κB/IκBα complex. The liberated NF-κB then translocates into the cell nucleus, where it binds to specific DNA sequences in the promoters of target genes, thus activating the transcription of pro-inflammatory cytokines and other inflammatory mediators [120]. Liu et al. [121] developed an injectable GelMA hydrogel modified with ZIF-8, which continuously releases Zn^2+^ to inhibit the NF-κB pathway and reduce the expression of inflammation-related genes, thereby promoting alveolar bone regeneration. Due to its injectability, it may serve as a potential bioink. The actions of IFN-γ and IL-4 are closely related to the activation of STAT (Signal Transducer and Activator of Transcription). IFN-γ promotes M1 macrophage polarization by activating the STAT1 signaling pathway, leading to increased PFKFB3-mediated glycolysis [122]. IL-4 promotes M2 macrophage polarization by activating the STAT6 signaling pathway. This pathway plays a crucial role in regulating the gene expression patterns associated with the M2 phenotype, contributing to anti-inflammatory and tissue repair functions [123]. In brief, research on signaling pathways related to macrophage polarization can guide the functionalization of GelMA. For example, understanding the impact of different signaling pathways on M1 and M2 macrophage polarization can help design functionalized GelMA bioinks to modulate cell polarization states and promote bone tissue regeneration.

#### 3.2.4 Improving the neuro-vascularized bone regeneration of GelMA

Natural bone tissue is a complex neuro-vascularized tissue and the process of regeneration and remodeling of bone tissue depends on neurovascular regulation and support. The periosteum covering the bone surface is richly distributed with blood vessels and nerves. The vascular network provides essential nutrients and growth factors for localized osteogenesis, whereas nerves activate and regulate the osteogenic process by sensing and transmitting changes in osteogenesis-related hormones (e.g., prostaglandin E2 and calcitonin, etc.) [124]. The nutrition and regulation of vascular neural network plays an important role in the reconstruction of large bone defects. However, at present, the role of the nervous system in bone tissue engineering has been largely ignored, so in the construction of bone repair scaffolders, the design of bionic bone layered structures that can induce neurovascular regeneration and osteogenesis is of great significance for promoting bone regeneration.

Electroactive materials have received much attention for their ability to promote the regeneration of electroactive tissues such as nerves, bone, and heart [125]. Currently, the nerve-inducing generative capacity of bio-scaffolds still relies heavily on the application of electrically conductive active materials. Among them, black phosphorus nanomaterials show promising applications due to their excellent electrical conductivity, good biodegradability, and excellent ability to promote neural differentiation [126].

In a recent study, Xu et al. [127] developed a bilayer hydrogel scaffold mimicking the structure of periosteum using magnesium ion-modified black phosphorus nanomaterials (BP) and GelMA hydrogel, which was capable of inducing neurovascular regeneration and bone regeneration simultaneously. They demonstrated that the scaffold significantly enhanced early angiogenesis and neurogenesis, ultimately promoting bone regeneration and remodeling *in vivo* and *in vitro*. In addition, Xu et al. [128] incorporated copper ion modified germanium phosphide (GeP) nanomaterial into GelMA hydrogel to prepare a multifunctional biological scaffold. The scaffold can not only significantly promote nerve differentiation and axon regeneration, but also continuously release copper ions to promote bone differentiation of BMSCs and angiogenesis of HUVEC while playing an antibacterial role. In another study, GelMA mixed with nerve growth factor (NGF) and Laponite was used to fabricate bioprinted structures that mimicked the central microenvironment of ossification and promoted nerve invasion. This bioprinted structure promoted the formation of functional bone tissue *in vivo* and *in vitro* by modulating the nervous system and vascularization and ossification [129]. Although some studies have demonstrated that the nervous system plays a key role in bone tissue engineering, and some useful attempts have been made to improve the neuro-vascularized bone regeneration ability of GelMA. However, there are still many questions that remain unanswered. For example, how to accurately reconstruct the bone, nerve, and blood vessel distribution similar to that of the natural bone so that they can better synergize with each other and so on.

#### 3.2.5 Developing personalized treatment strategy of GelMA for bone regeneration

It is hoped that the application of bioprinting in bone tissue regeneration can achieve ideal personalized repair. Therefore, in addition to using bioprinting technology to customize the scaffolds with specific bone defect shape of patients, it is more important to focus on the biological function of the printed structure to provide specific biological clues, such as growth factors.

Allogeneic growth factors require complete decellularization. Incomplete decellularization is prone to the risk of immune rejection and disease transmission, but complete decellularization reduces biological activity [130]. At present, there are also some beneficial attempts on acellular matrix hydrogel bioinks. Yang et al. [131] prepared bioink by combining decellularized extracellular matrix(dECM) from porcine tooth hair follicles with GelMA. The addition of dECM provided a beneficial peridental microenvironment for loaded periodontal stem cells, which could induce periodontal tissue regeneration. In addition, in a recent study, Gao et al. [132] incorporated acellular pig bone meal into GelMA for bone tissue engineering scaffolds. They demonstrated that the scaffold could induce osteogenic differentiation of human BMSCs even in the absence of an induction medium.

Compared with allogeneic growth factors, autologous growth factors have significant advantages such as high biosafety, low exclusion and excellent effect, but the sources are relatively limited. There are attempts to use patient-specific biological cues to promote bone regeneration of printed structures. Patient-specific biological cues have some obvious advantages like being readily available, avoiding immune rejection, and reducing the ethical regulatory burden of clinical transformation and so on. Currently, platelet rich plasma (PRP) and patient-derived bone particles (BPs) have been used to prepare patient-specific bioinks based on GelMA hydrogels, respectively. Patient-derived PRP can provide abundant autologous growth factors for printed structures and promote stem cell recruitment, osteogenesis and vascular formation [116]. While the presence of BPs can not only enhance the mechanical properties of the printed structure, but also provide living cells and an already formed complete mineralized matrix, which is all conducive to osteogenesis [133]. Although PRP and BPS have shown excellent patient specific bone repair effect, the exploration on the source of patient specific biological clues is still very limited, which is an urgent problem to be solved in personalized bone repair.

## 4 GelMA functionalization strategies with smart stimuli-responsive

The so-called “smart response” refers to that biomaterials can flexibly, quickly identify and respond to external stimuli or internal disease microenvironment changes, so as to enhance bone regeneration and bone therapy effects [134]. According to the latest definition of “smart biomaterial” proposed by Montoya et al. [135],"smartness” is summarized as having four levels: inert, active, responsive, and autonomous. “Smart” in this part refers to GelMA hydrogel's ability to be active, responsive, and even autonomous in the face of various biological environmental changes.

**Fig. 13:**
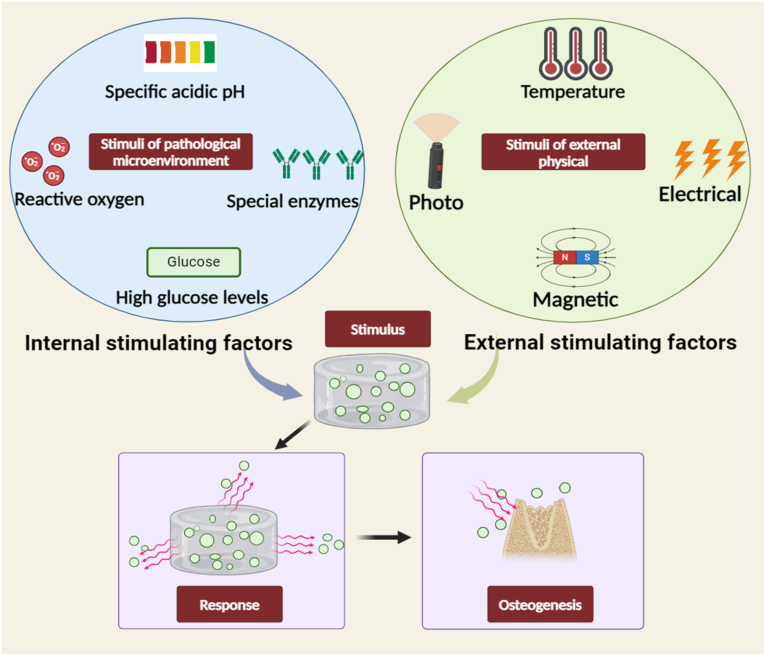
GelMA hydrogels promote osteogenesis in response to different types of external and internal stimulating factors.

In recent years, smart responsive GelMA hydrogel has been studied in the field of bone regeneration. In this section, different stimulus-response strategies of GelMA hydrogel are briefly reviewed, which include internal pathological microenvironment stimulus-response strategies (such as reactive oxygen species, pH, specific enzymes, and high sugar) and external physical stimulus-response strategies (thermal, electrical, optical, magnetic and mechanical) (Fig. 13).

### 4.1 Internal pathological microenvironment stimulus response strategies

**Fig. 14:**
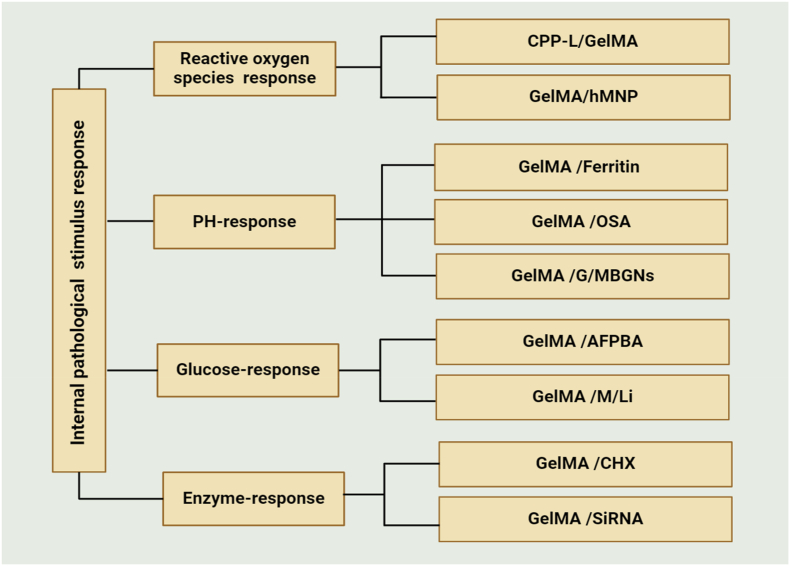
Schematic diagram of GelMA response strategies to internal pathologic microenvironmental stimuli.

GelMA hydrogel has shown good prospects for bone regeneration in the microenvironment of pure bone defects. However, most bone defects are caused by various diseases which have special pathological microenvironments. For example, excessive reactive oxygen species [136] and specific acidic pH [137] are produced in tumors and inflammation, special enzymes produced by bacteria in severe infections [138], and high glucose levels in diabetic bone defects [139]. In a word, bone regeneration in diseases such as inflammation, tumor, and bacterial infection still faces great challenges. Therefore, functional GelMA hydrogels that respond to internal microenvironmental stimuli are very important for activating bone disease treatment and regeneration (Fig. 14).

#### 4.1.1 Reactive oxygen species-response strategies

Reactive oxygen species (ROS) refer to peroxide, superoxide, hydroxyl radical and other oxygen metabolism chemicals [140]. When the body is injured and diseases such as inflammation and tumor occur, ROS will accumulate in bone defects. Excessive ROS will promote the generation of oxidative stress, leading to the degradation of mineralized matrix and bone metabolism [95]. Therefore, in the treatment and regeneration of bone diseases, it is particularly important to prepare responsive biomaterials with ROS scavenging ability.

To prepare ROS-responsive antioxidant scaffolds, Sun et al. [141] developed a ROS-responsive oxygen-releasing nanoparticle system encapsulated with catalase in liposomes (CPP-L) and then functionalized GelMA. The CPP-L/GelMA hydrogel acts as a microenvironment regulator during bone tissue respiration, delivering catalase to hydrolyze ROS in the hypoxic environment of bone defects, generating oxygen. Additionally, excess ROS triggers a hydrophilic change in Poly (propylene sulphide) (PPS) located on the surface of oxygen-carrying nanoparticles in the hydrogel, releasing oxygen to further improve oxygen supply as needed. This oxygen-rich microenvironment promotes angiogenesis, suppresses osteoclast formation, enhances BMAL1 gene expression in osteoblasts, and facilitates osteogenic differentiation through enhanced autophagy, significantly promoting bone regeneration. In another study, Li et al. [142] engineered GelMA with hollow manganese dioxide nanoparticles (hMNPs) that can degrade H_2_O_2_ and produce oxygen. *In vitro*, these hMNPs eliminated intracellular ROS, safeguarding cells from oxidative harm while stimulating cell growth through oxygen release. *In vivo*, GelMA/hMNP composite hydrogels responded dynamically to changes in bone microenvironment ROS levels by releasing oxygen and bone morphogenetic protein-2 (BMP-2)-related peptides as needed.

The ROS responsiveness of GelMA is mainly obtained by changing the conformation of the scaffold in the oxidative microenvironment. Functionalized GelMA scaffolds are loaded with antioxidants that can be released and activated in the oxidative environment and used to scavenge excess ROS. Based on this, ROS-responsive scaffolds can also be used for drug delivery. GelMA scaffolds loaded with antioxidants and one or more drugs undergo redox reactions to change their structure in an oxidizing environment, thus achieving targeted drug release [140].

#### 4.1.2 The pH- response strategies

As we know, the normal human microenvironment is weakly alkaline (pH = 7.4) [143]. When there is inflammation, infection, or tumor in the body, the pH of this tissue is 0.5–1 pH units lower than that of surrounding healthy tissue [137]. This slightly acidic pathological microenvironment can be used to prepare responsive GelMA-based biomaterials with bone healing and regeneration effects. Yao et al. [144] added oxidized sodium alginate (OSA) into GelMA to prepare a pH responsive dual-network GelMA-OSA hydrogel scaffold. In this converts light energy into gentle heating (40 °C – 43 °C) scaffold, OSA with aldehyde group reacts with GelMA with amine group to form the Schiff-base bond (-C=N-). Under acidic conditions of bacterial infection, the Schiff-base bonds in GelMA-OSA are reverentially broken, and the gentamicin sulfate (GS, an antimicrobial agent) and phenylalanine (Phe, a small molecule activator of BMP2) carried in GelMA are released, acting as antibacterial and osteogenic agents. Ferritin has pH-dependent assembly properties and can be loaded with various substances (drugs, DNA, etc.) and released in response to specific pH changes, enabling pH-responsive targeted drug delivery systems. Samanipour et al. [145] designed and prepared a functional GelMA hydrogel conjugated by ferritin nanocages. The covalent binding of ferritin on the GelMA network not only made it have pH responsive but also increased the compression modulus of the GelMA hydrogel by about 4 times, which was conducive to bone formation.

In addition to using acidic pH as a responsive stimulant that triggers the release of the drug GelMA carries to achieve anti-inflammatory and anticancer effects. The pH of the *in vivo* microenvironment also plays an important role in bone repair, and the osteogenic activity of mesenchymal stem cells was significantly enhanced when pH increased from 6.6 to 7.8 [146,147]. Therefore, correcting the acidic microenvironment is very important for bone regeneration. Xin et al. [148] crosslinked GelMA with mesoporous bioactive glass nanoparticles (MBGNs) to prepare GelMA-G-MBGNS artificial periosteum. With the release of silicon ions in the hydrogel, the acidic microenvironment of local body fluids was corrected and the pH was stable at about 7.55, creating a more favorable microenvironment for bone regeneration.

#### 4.1.3 Glucose-response strategies

In recent years, with the increasing prevalence of diabetes, bone defect repair for diabetic patients has also attracted the attention of scholars [149,150]. Previous studies have shown that high blood glucose levels in patients with diabetes can cause an increase in reactive oxygen species, resulting in bone defects that remain in a pro-inflammatory microenvironment, which is not conducive to bone regeneration [139]. To better promote bone regeneration in diabetic patients, some research groups have used blood glucose concentration as a stimulus to design GelMA hydrogels with glucose-responsive functions to deliver insulin or some growth factors, for example.

In a recent study, Guo et al. [151] prepared a glucose-responsive GelMA hydrogel using in situ copolymerization of the glucose-responsive monomer 4-(2-acrylamidoethylcarbamoyl)-3-fluorophenylboronic acid (AFPBA) and glucose-insulin with GelMA hydrogel. When in a high glucose environment, the phenylboronic acid moiety in the GelMA hydrogel binds preferentially to the diol group in glucose, thereby releasing the originally carried G-insulin. In addition, Wu et al. [152] developed lithium (Li)-modified bioglass-GelMA scaffolds (GM/M - Li). Lithium ions can be continuously released in the hyperglycemic state to reduce inflammation by modulating macrophage-stimulated immunomodulatory function while secreting bone morphogenetic protein-2 (BMP-2) and vascular endothelial growth factor (VEGF) to promote osteogenesis and vascular regeneration in the hyperglycemic microenvironment.

Currently, there are fewer studies on glucose response strategies for GelMA, and there is still a long way to go in the future. In addition to developing GelMA hydrogels with dual functions of blood glucose control and bone formation promotion, future research should also focus on improving the sensitivity of blood glucose response of GelMA hydrogels, that is, sensitively sensing the fluctuation of blood glucose in the microenvironment, making timely response according to pathological blood glucose changes, and creating a microenvironment suitable for bone tissue regeneration.

#### 4.1.4 Enzyme-response strategies

Enzymes are highly specific and selective active molecules, which play an important role in bone growth, absorption, occurrence and development of bone diseases and other biological processes [138]. Based on this, many researchers use enzymes as biological triggers to develop GelMA hydrogels with intelligent responsiveness.

Matrix metalloproteinases (MMPs) are the main enzyme family responsible for extracellular matrix remodeling [153]. Previous studies have shown that MMPs are specifically overexpressed in osteoarthritis and osteoporosis. More importantly, GelMA has MMPs sequence, which will degrade in high levels of MMPs. Therefore, GelMA can be used to develop drug carriers that are responsive to MMPs. In a recent study, Ribeiro et al. [154] used GelMA to prepare a hydrogel that releases chlorhexidine (CHX) on demand. When GelMA is exposed to high levels of matrix metalloproteinases, it will release CHX and play an antibacterial role. Similarly, Cai et al. [155] prepared MMPs-responsive GelMA hydrogels loaded with siRNA nanoparticles by microfluidic techniques, and released siRNA nanoparticles after the elevation of MMPs-2 to exert anti-adhesive effects and successfully prevent adhesion of new tissues. However, at present, no scholars have further combined MMPs responsive GelMA hydrogels with drugs or factors that promote bone formation. The disease treatment and osteogenic effect of MMPs responsive GelMA in osteoarthritis and bone tumors need to be further studied.

In addition to reactive oxygen species, pH, glucose, and specific enzymes, there are other specific pathological microenvironments. For example, specific ion concentrations, like high calcium ion levels in the presence of bone tumors [156], and endogenous electronegative potentials present in bone defects [157], which can serve as signal sources for GelMA hydrogel stimulation responsive. Although currently, there are fewer studies on these specific signal responsive strategies for GelMA in the field of bone regeneration, they also provide ideas for scholars to develop specific responsive scaffolds in the future.

### 4.2 External physical stimulus response strategies

**Fig. 15:**
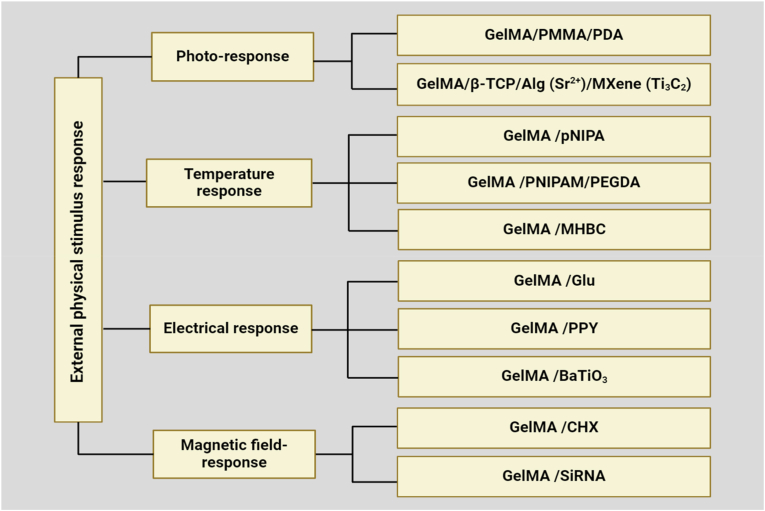
Schematic diagram of GelMA response strategies to external physical stimuli.

In addition to internal pathological stimuli caused by diseases of the body, artificially imposed external physical stimulation, such as light, electrical, magnetic and temperature can also change the internal structure and function of GelMA hydrogel. Therefore, many researchers have worked to develop GelMA hydrogels that can respond to external physical stimuli to further achieve spatial and temporal control of GelMA functions, such as triggering the release of drugs at a certain point in time or promoting osteogenic differentiation of cells, so as to achieve more desirable bone regeneration and bone therapy effects (Fig. 15).

#### 4.2.1 Photo-responsive strategies

Photo-responsive hydrogels contain light responsive group components, which can transform light signals into various physical signals, so as to change the physical or chemical properties of hydrogels, dynamically guide cell behavior, and control local microenvironment [158]. Photo-responsive hydrogels are divided into photo-crosslinked and photo-degradable hydrogels, and as we know, GelMA is one of the photo-crosslinked hydrogels. In addition to giving GelMA a stable morphology, photo-responsive has many promising applications in bone regeneration and bone therapy.

**Fig. 16:**
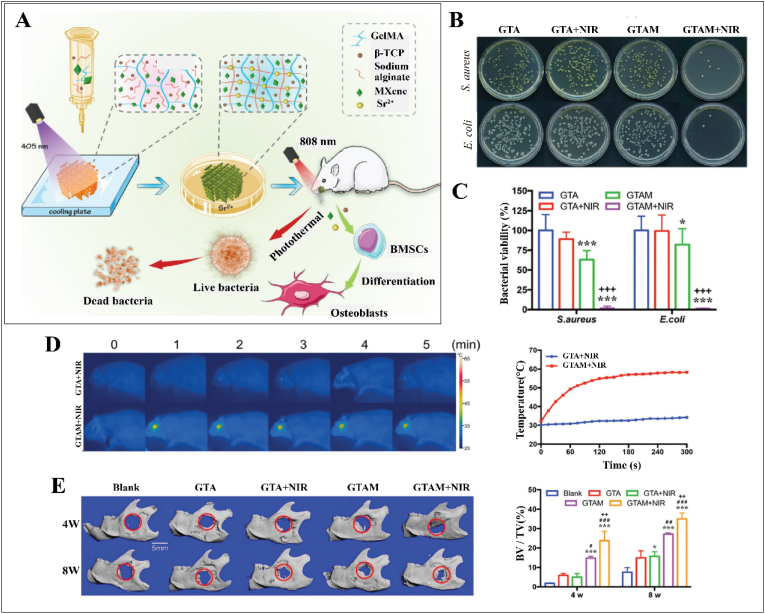
A）Schematic illustration of the 3D printing procedure of MXene composite hydrogel scaffolds, and photothermal antibacterial activity and bone regeneration in infected mandible defect models. B）Images of *S. aureus* and *E. coli* clones co-cultured with 3D printed scaffolds after 24 h, then with or without NIR. C) Bacterial viability in images of *S. aureus* and *E. coli* clones. D) Infrared thermal images, and photothermal heating curves of 3D printed scaffolds implanted into the mandibular defects of rats. E) Micro-CT 3D reconstruction images of rats with infected mandibles after 4 weeks and 8 weeks of scaffold implantation, and value of BV/TV of new-born bone tissue in the defect area. (*p < 0.05 ***p < 0.001 compared to GTA, p < 0.001 compared to GTAM.) [reprinted with permission from Nie et al. [161]; copyright (2022) Nanoscale].

Photothermal therapy (PTT), which converts light energy into gentle heating (40 °C – 43 °C) by means of a light absorber, has shown great potential in promoting bone regeneration [159]. Based on this, Wu et al. [160] prepared GelMA/polymethylmethacrylate (PMMA)/polydopamine nanoparticles (PDA) photo-responsive hydrogels. PDA is a kind of photothermic agent, which can gently convert light energy into heat energy under 808 nm laser irradiation. Their results showed that this composite light responsive hydrogel showed excellent osteogenic effect *in vitro* and *in vivo* skull defect repair. In another study, Nie et al. [161] developed a composite bioink comprising GelMA/β-TCP/sodium alginate (Sr^2+^)/MXene (Ti_3_C_2_) (GTAM). The MXene conferred excellent photothermal properties to this bioink, generating heat under near-infrared irradiation and thereby killing bacteria. Additionally, the GTAM bioink exhibited osteogenic differentiation effects. Ultimately, this dual-functional bioink loaded with BMSCs in a bioprinted scaffold promoted the regeneration of infected mandibular bone defects (Fig. 16).

Despite some encouraging results have been obtained regarding photo-responsive GelMA hydrogels, there are still some problems, such as the limited penetration depth of light, which need to be further addressed in the future.

#### 4.2.2 Temperature response strategies

Temperature-responsive hydrogels are able to respond to temperature changes and affect the expansion and contraction of the hydrogel, and such changes are reversible and have promising applications in controlling the release of drugs, cells, enzymes, etc [162]. GelMA hydrogel is also a temperature-sensitive hydrogel, but after photo-crosslinking, it will acquire an irreversible steady state and lose its temperature-sensitive properties. Nevertheless, there are still many functionalization strategies that can re endow GelMA with temperature response characteristics.

Aldana et al. [163] used GelMA and polylactic acid (pNIPA) to prepare a temperature responsive interpenetrating network hydrogel (NPxG). Due to the temperature sensitivity of polylactic acid, this hydrogel can selectively adhere/separate cells on the scaffold according to temperature changes. Similarly, Dabiri et al. [164] prepared a multifunctional temperature responsive microcarrier composed of GelMA/poly (N-isopropylacrylamide) (PNIPAM)/polyethylene glycol diacrylate (PEGDA). This temperature-responsive microcarrier allowed cells to grow stably attached on the surface of the microcarrier at 37 °C. When the temperature was reduced to room temperature, the cells were separated from the surface of the microcarrier. In conclusion, they believe that this microcarrier can obtain cells by non-invasive heat induction without using proteolytic enzymes, and has great application prospects in the delivery of therapeutic cells, growth factors, drugs and so on in the future. In addition, Luo et al. [165] prepared thermal/optical dual cross-linked hydrogels with GelMA and butyl methacrylate chitosan (MHBC), a thermosensitive material that undergoes uniform and reversible volume contraction of the hydrogel with increasing temperature. GelMA, on the other hand, provides biocompatibility to the composite hydrogel and promotes cell adhesion ability. They believe that this reversible shrinkage is expected to be used to fabricate high-resolution microstructures.

The above studies show that GelMA temperature response has great potential for drug release and preparation of microstructures, but has not yet been validated *in vivo*. Considering the special constant-temperature environment *in vivo*, to realize temperature response in constant-temperature environment, it is necessary for GelMA to have a very high temperature sensitivity, which can respond to a small range of temperature changes. The realization of fine temperature regulation and flexible temperature control technology is the future direction of GelMA temperature response. Although the relevant research of GelMA hydrogel temperature response strategy has not been applied in the field of bone regeneration, these have laid a foundation for future research. In the future, it is expected to use GelMA composite hydrogel with temperature response to selectively release the carried therapeutic drugs in time and space, or promote the active molecules of osteogenesis, so as to obtain a more ideal effect of bone disease treatment and bone regeneration.

#### 4.2.3 Electrical response strategies

**Fig. 17:**
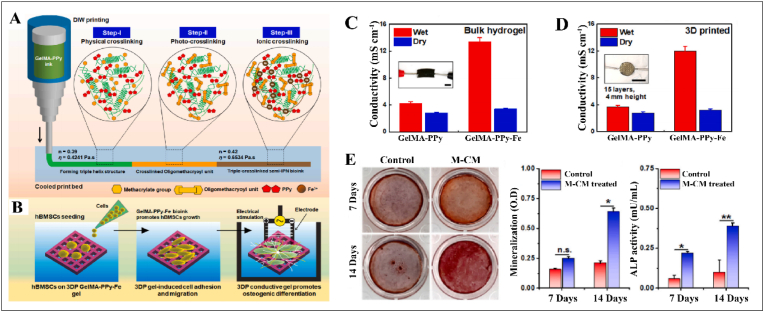
A）Schematic illustration for the fabrication process of 3D printed triple-crosslinked GelMA-PPy based hydrogel for improving the conductivity and bioactivity. B) Schematic illustration of direct current stimulation for regulating stem cell proliferation and osteogenic differentiation. C) Conductivity of the GelMA-PPy and GelMA-PPy-Fe hydrogels in wet and dry form. D) Conductivity test of the 3D printed GelMA-PPy and GelMA-PPy-Fe hydrogels. E) ARS staining of hBMSCs in the presence of M-CM after 14 days culture. Quantification of ARS and ALP activity of the hBMSCs in the presence of M-CM. [reprinted with permission from Dutta et al. [169]; copyright (2023) Biomaterials].

Previous studies have shown that electrical stimulation can increase the osteogenic potential of various types of mesenchymal stem cells and osteoblast-like cells and stimulate bone, cartilage, and vascular tissue formation [166,167]. Combining electroactive materials with biomaterials to produce “smart” biomaterials with electrical stimulation response allows for more precise regulation of cellular activity, drug delivery to achieve better bone regeneration and therapeutic effects [168]. GelMA hydrogels do not possess electrical conductivity but can be combined with conductive polymers such as polypyrrole (PPY) [169], polyaniline (PANI) [170], etc. to obtain electrical stimulation response properties. Recently, Bansal et al. [171] used GelMA and polypyrrole to form a hybrid hydrogel for the electrically controlled release of glutamate (Glu) to achieve controlled drug release in response to electrical stimulation. Bone collagen has been reported to possess piezoelectric properties, resulting in the bone microenvironment having an endogenous electric field (EnEF) [172]. Conductive bioinks, acting as the medium for transmitting electrical signals, have been shown to enhance cell communication and promote osteogenesis in the presence of endogenous electric fields [173]. However, in many studies, external electric fields are applied to facilitate bone regeneration. GelMA, lacking inherent conductivity, can be made conductive by incorporating conductive materials. By adjusting the composite ratio of these materials, bioinks with electrical conductivity close to that of natural bone can be obtained, thereby promoting bone regeneration. Dutta et al. [169] introduced Fe ions into PPY-grafted GelMA to adjust the electrical conductivity of the bioink, making it approximate to cortical bone (∼0.2 mS cm^−1^) and trabecular bone (∼0.79 mS cm^−1^) (Fig. 17).

Piezoelectric hydrogels are another common type of electrically responsive bioink. The piezoelectric effect allows them to convert mechanical force into electrical stimulation, making them particularly suitable for bone defects in areas under stress, such as alveolar bone and limb bones. Liu et al. [174] prepared a nanocomposite hydrogel composed of piezoelectric tetragonal BaTiO_3_ nanoparticles and GelMA. Under mechanical stimulation, this hydrogel triggers electrical stimulation, improving the mitochondrial bioenergetic function of periodontal ligament stem cells (PDLSCs) and rescuing their osteogenic differentiation.

Although the application of electrically responsive GelMA bioinks in bone regeneration is currently limited, the above research base indicates that GelMA electrical stimulation response has excellent potential for application in the field of bone regeneration, and it is believed that there will be a breakthrough in the near future. In addition, in using electric current as a stimulating factor, care should be taken to avoid problems such as excessive current stimulation, which produces local heating causing damage to cells.

#### 4.2.4 Magnetic field-response strategies

Magnetically responsive GelMA hydrogels are mainly composed of GelMA and magnetic particles, which respond to external magnetic fields, thus changing the structural changes of the hydrogel and remotely modulating the biochemical properties of cells, tissues [175]. Jalili et al. [176] prepared magnetothermal dual-responsive hydrogels loaded with dexamethasone (DOX) using N-isopropylacrylamide-co-acrylamide (PNIPAM) and GelMA, which exhibited temperature and magnetic field-dependent release of DOX, and they investigated the *in vitro* efficacy of DOX using osteoblasts and osteosarcoma cells also. They demonstrated that on-demand local delivery of drugs or active molecules could be achieved by magnetothermal activation. Since the magnetic stimulation signal is not affected by tissue depth, it has great advantages in the treatment of bone lesions which in deep tissue. However, some magnetic nanoparticles with small diameter (<50 nm) can penetrate the biofilm, cause tissue inflammation and induce cell apoptosis [177]. Therefore, the size and dosage of magnetic nanoparticles should be considered in the future research also.

In short, compared with the internal pathological stimulation of the body itself, the artificially imposed external physical stimulation has higher controllability and is an ideal choice for drug delivery system. However, there are still few studies on GelMA stimulation responsive hydrogels in the field of bone regeneration and bone therapy, and there are some corresponding limitations, which need further exploration and research.

## 5 GelMA functionalization strategies with dual function of bone disease treatment and bone regeneration

**Fig. 18:**
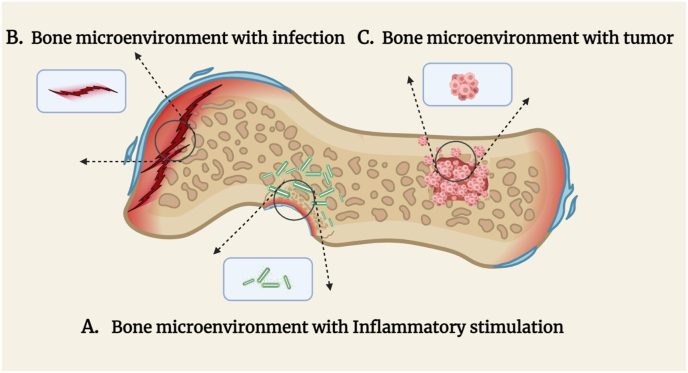
Schematic diagram of the pathological bone defect environment with infection, inflammation, and tumor.

Bone defects caused by diseases such as infection, inflammation (osteomyelitis) and tumors, in addition to surgical intervention, require further treatment, control and prevention of the disease during subsequent bone regeneration and repair therapy to ensure that the desired bone repair outcome is achieved [178]. Therefore, bone regeneration in diseases such as inflammation, tumor, and bacterial infection still faces great challenges (Fig. 18). In this section, we briefly reviewed the bone regeneration strategies of GelMA in these three common diseases.

### 5.1 GelMA with both anti-inflammatory and bone regeneration functions

Inflammation, regeneration, and remodeling are the three stages of bone tissue regeneration [179]. Brief inflammatory stimuli can create a regenerative microenvironment that contributes to bone and angiogenesis, which is essential for bone tissue repair. However, dysregulated, persistent and severe inflammation hinders bone tissue regeneration [180]. The use of immunomodulation to convert pro-inflammatory to anti-inflammatory modulation mentioned in the previous section is limited and the use of anti-inflammatory drugs is the most rapid and effective approach when the organism is in a more severe inflammatory state. Currently, combining anti-inflammatory therapies into GelMA to effectively promote bone regeneration remains a challenge, with a focus on the stable and sustained delivery of anti-inflammatory agents to the site of bone repair.

Zhu et al. [181] took advantage of the electrostatic interaction between the natural amino group of chitosan (CS) and carboxyl group to prepare a GelMA/CS double network hydrogel scaffold encapsulated with aspirin (ASA). ASA was well wrapped in the hydrogel due to the electrostatic interaction between CS and ASA, showing a sustainable local sustained-release behavior. They elucidated the dose-dependent bone treatment and regeneration efficiency of the stent by promoting anti-inflammatory treatment and osteogenic differentiation. Aluminosilicate nanotubes (HNT) are often used as a drug delivery vehicle due to their special bilayer hollow tubular structure (positively charged inner surface and negatively charged outer surface). Bordini et al. [182] prepared scaffolds by combining nanosilicates loaded with dexamethasone, a drug with anti-inflammatory and mineralizing effects, with GelMA, and they demonstrated that this scaffold could slowly release dexamethasone (DOX) under inflammatory conditions and trigger the regenerative capacity of mineralized tissue in situ. In a recent study, Du et al. [183] utilized manganese silicate (MS) nanoparticles to functionalize GelMA hydrogels, creating a bioink with anti-inflammatory and immunomodulatory functions. Subsequently, they used multicellular bioprinting to construct a tendon-bone composite *in vitro*, which suppressed local chronic inflammation and facilitated tendon-to-bone regeneration.

In addition, GelMA hydrogel microspheres are also an effective drug delivery carrier because of their structural stability and flexibility, which can realize the local release of drugs. In a recent study, Han et al. [184] successfully used GelMA microspheres to carry the anti-inflammatory drug diclofenac sodium (DS), and the slow release of DS at the joint site showed good therapeutic effects in osteoarthritis.

### 5.2 GelMA with both antibacterial and bone regeneration functions

Bacterial infections that are easily caused by either stenting procedures or the presence of trauma sites. Bacterial infections can lead to prolonged inflammation, progressive bone destruction, and thus impede bone regeneration [185]. Therefore, the development of bifunctional GelMA-based scaffolds with antibacterial and osteogenic functions has become imperative. Previous studies have shown that typical pathogens of orthopedic surgery-associated infections include Gram-negative and Gram-positive bacteria such as: *Staphylococcus aureus*, *Escherichia coli*, *Pseudomonas aeruginosa*, and *Staphylococcus epidermidis* [186].

At present, the research and development of antibacterial scaffolds are mostly focused on metal ion biomaterials. Studies have found that these metal cations released into body fluids have good bone promoting and antibacterial functions. For example, bioactive glass, silver ion [117], strontium ion [187] and lithium ion [188] have been proved to have good antibacterial activity. Moghanian et al. [186] prepared a GelMA/BG-5/5 composite scaffold by incorporating modified strontium and lithium doped bioactive glass (BG-5/5) into GelMA hydrogel. Strontium ions released from the scaffold, and lithium ions have good bactericidal activity and osteo-inductive effect on *S. aureus*, and have significant potential in the treatment of orthopedic infections. In a previously mentioned study, the nanosilver/aluminosilicate nanotube/GelMA scaffold prepared by Ou et al. [117] is a scaffold that combines both bone immunomodulatory and antimicrobial activities. They demonstrated that the nanosilver in the scaffold has broad-spectrum antimicrobial properties and low toxicity, with proliferation inhibition against E. coli and *S. aureus*, and demonstrated the antimicrobial and bone-enabling effects of the scaffold in infected bone defects by *in vivo* experiments. In a recent study, a composite scaffold made of Zeolitic imidazole framework-8 and GelMA had a significant antibacterial effect on porphyromonas gingivalis due to the sustained release of zinc ions from ZIF-8 nanoparticles, which not only reduced the bacterial load in periodontal pockets but also effectively promoted alveolar bone regeneration [189].

Although the strategy of functionalizing GelMA with various metal ion materials has been proved to have good antibacterial and bone promoting effects, antibacterial metal ions also have some limitations, such as the toxicity of high concentration of silver ions to eukaryotic cells. Therefore, how to ensure the safe ion concentration and develop other antibacterial strategies still need to be further explored. In the aforementioned study, Nie et al. [161] imparted photothermal properties to GelMA by adding MXene. Under near-infrared irradiation, the bioprinted scaffold carried out photothermal therapy (PTT) to kill bacteria, ultimately achieving the repair of infected mandibular bone defects.

### 5.3 GelMA with both anti-tumor and bone regeneration functions

Bone tumors are a major cause of bone tissue destruction and loss, among which osteosarcoma is one of the most common malignant tumors of the skeletal system [190,191]. Conventional tumor treatment methods include surgical resection, chemotherapy and radiotherapy. However, surgical resection results in large bone defects and there are still residual tumor cells with the risk of recurrence, bacterial infection, etc [192]. In recent years, with the continuous development of biomaterials science, bone therapeutic bio-scaffolds based on biomaterials combined with anti-tumor drugs have shown excellent application prospects [160]. Therefore, the development of bifunctional scaffolds with bone tumor treatment and bone regeneration is particularly important. The ideal bone treatment scaffold is expected to inhibit tumor growth in the early stage of treatment and enhance bone tissue repair in the late stage of treatment.

Recently, photothermal therapeutic biomaterials have shown good bone tumor treatment and bone regeneration effects in the treatment of deep tumors and are a current research hotspot for antitumor scaffolds [189,193]. Based on this, Yin et al. [194] developed a GelMA scaffold modified by MXene nanosheets and sulfonated polyetheretherketone (SP), which could have the ability to kill wounded osteosarcoma cells under near-infrared irradiation using the synergistic photothermal effect of MXene and polydopamine. In addition, they also loaded tobramycin (TOB) on the scaffold, and the SP@ MX-TOB/GelMA scaffold showed strong antibacterial properties, and more importantly, the scaffold also had good osteogenic ability and osteointegration ability. They concluded that the scaffold simultaneously combines multiple functions such as anti-tumor, antibacterial and osteogenesis promotion, providing a promising countermeasure for the treatment of histopathology and bone regeneration after resection of osteosarcoma. Liao et al. [195] used gold nanorods, nanohydroxyapatite, chondroitin methacrylate sulfate, and GelMA to prepare a hybridized hydrogel scaffold with photothermal effect. The photothermal ability of this scaffold hydrogel can induce apoptosis of tumor cells remaining after surgery, thus eradicating the residual tumor after surgery, and the scaffold can occupy the tissue-deficient area, which helps bone regeneration. This bifunctional hybrid hydrogel scaffold with the ability to prevent tumor recurrence and promote bone formation provides a new idea for the treatment of complex osteosarcoma.

In addition to photothermal therapy, the use of GelMA hydrogels loaded with antitumor drugs to provide sustained drug release in the lesion area is also a local treatment for cancer. In a recent study, Wu et al. [196] used GelMA-loaded liposome-modified gemcitabine to provide good inhibition of osteosarcoma in mice carrying MG63 tumor cells by slow release of the drug locally in the tumor.

Many GelMA hydrogel-based modification strategies have been applied to cancer-related prevention and treatment, although most of them have not yet been combined with bone regeneration functions, but this also lays the foundation for the development of GelMA scaffolds with both anti-cancer and bone regeneration functions, on which future scholars can conduct more research.

## 6 GelMA functionalization strategies with endogenous stem cell recruitment function

Another great challenge for bone tissue engineering is the low survival rate of locally transplanted cells. Low cell survival at the site of bone defects can lead to problems such as osteogenic differentiation within the structure and insufficient vascularization [197]. Wang et al. [198] have previously demonstrated lower long-term survival of cells in bioprinted GelMA scaffolds compared to poured GelMA scaffolds. In addition, they also proposed in another study that *in vitro* and *in vivo*, the long-term cellular activity of cells in high concentration (10 %) GelMA bioprinted scaffolds was lower than that in low concentration GelMA bioprinted scaffolds (3 %) [199]. These studies indicate that the bioprinting process and different bioprinting parameters can adversely affect the cellular activity of the loaded cells. For this reason, the development of scaffolds with endogenous stem cell recruitment has gradually become a hot research topic. The ideal GelMA-functionalized scaffold can induce the directed homing of endogenous stem cells in the defective tissue and provide a suitable microenvironment for osteogenic differentiation of endogenous stem cells, thus achieving sustained good bone tissue regeneration.

At present, the most studied method of stem cell recruitment is through local delivery of growth factors to provide guidance signals for the recruitment of endogenous stem cells. Among these, stromal cell-derived factor 1-α (SDF-1α) has been shown to direct the homing and migration of BMSCs [200]. Based on this, Shi et al. [201] prepared a GelMA–SN–SDF-1α hybrid scaffold using SDF-1α, silicate nanoplatelets (SN), in which SDF-1α has the effect of inducing stem cell homing，which can attract BMSCs to the defect area, while SN promotes BMSCs to osteogenic differentiation in the absence of osteo-inductive factors. In conclusion, the scaffold showed good ability to repair bone defects in a rat cranial defect model. In addition, previous studies have shown that both VEGF-A [202] and BMP-2 [203] have functions in directing the recruitment of undifferentiated stem cells, which, although not yet validated in GelMA hydrogel scaffolds, may also provide ideas for designing future GelMA functionalization strategies.

In addition to growth factors, another way to induce stem cell recruitment and osteogenic differentiation is to load polypeptides with chemical attraction properties in GelMA. In a study by Huang et al. [204], ECM was modified with bone marrow homing peptide sequence PFS (PFSSTKT), and PFS ECM particles were added to GelMA hydrogel to prepare scaffolds. They showed that after GelMA/ECM PFS stent was implanted into rabbits, PFS simulating bone marrow homing peptide could promote the recruitment of endogenous BMSCs at the defect site, thereby promoting cartilage repair.

In conclusion, the endogenous stem cell recruitment functionalization strategy of GelMA can compensate the shortcomings of insufficient number and activity of exogenous stem cells and further promote osteogenesis to accelerate osseointegration.

## 7 Conclusions and future perspectives

**Fig. 19:**
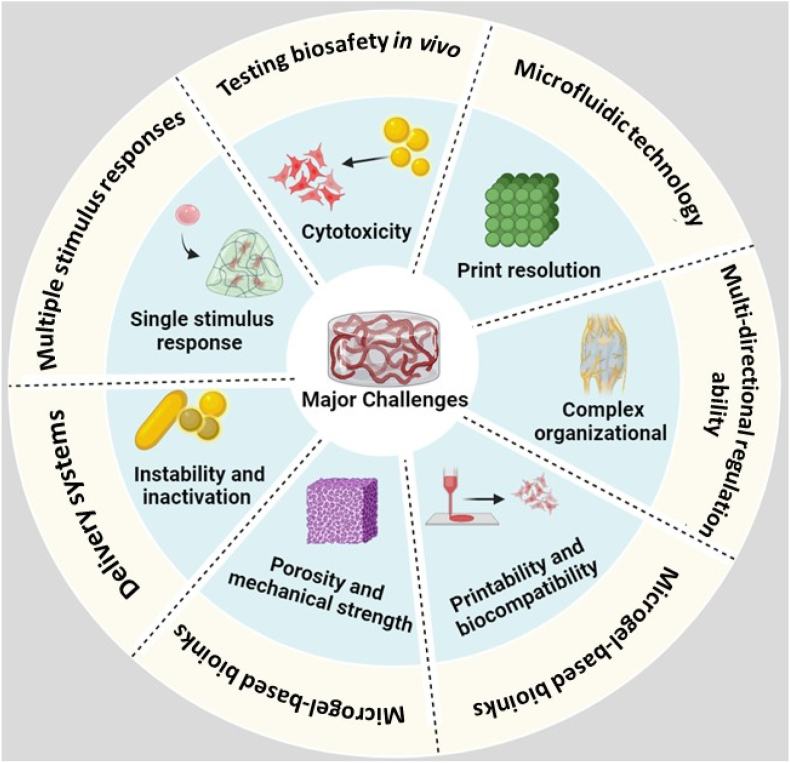
Schematic of the main challenges and possible solutions of GelMA bioinks for bone tissue regeneration.

Currently, 3D bioprinting has attracted significant attention as one of the most promising manufacturing processes for bone tissue engineering and bone regeneration. There are already commercial GelMA-based bioinks entering the bioprinting market, such as BioGel (Biobot, USA) and Gel4Cell® (Bioink Solutions Inc, South Korea). However, most commercial bioinks are still based on single component bioinks. As one of the main irreplaceable parts of bioprinting, the development of bioink is crucial for its application in bone tissue engineering. Herein, we briefly introduced the requirements of bioinks for bone regeneration and discussed in combination with the properties of GelMA hydrogel. Especially, the functionalization strategies developed for the mismatch properties offered by GelMA hydrogel were emphasized. In addition, the response strategies of GelMA hydrogel to external physical stimulation and internal pathological microenvironment stimulation, as well as the functionalization strategies of GelMA hydrogel to achieve both disease treatment and bone regeneration in the presence of various common diseases (such as inflammation, infection, tumor) are also briefly reviewed. Although significant progress has been achieved, GelMA-based bioinks remain impractical for clinical translation (Fig. 19).

(1) Although the biosafety of GelMA has been verified, the functionalized GelMA bioinks need to be further investigated for their long-term *in vivo* degradation and cytotoxicity due to the incorporation of nanomaterials or metal ions.
(2) Current researchers have made a conscious effort to improve the printability and vascularization of bioinks, however, there is still a need to improve the print resolution to print accurate internal structures to incorporate the vascular network. Some methods, such as microfluidics, have been incorporated into bioprinting techniques to improve the resolution of bioprinting. The development of bioinks with higher printing resolution is still needed to reproduce the structure of natural bone in the future.
(3) There is a conflict between the porosity of the bioinks and the mechanical strength of the printing structure. Increasing the porosity of GelMA hydrogel is beneficial to cell survival and angiogenesis, but it will reduce the mechanical strength of printing structure. Microgel-based bioinks may serve as a balanced solution. This bioink is composed of micron-sized hydrogel microspheres that are cross-linked together on their surfaces after extrusion. This approach allows to separate the cellular microenvironment from the scaffold so that it can be regulated separately to achieve equilibrium.
(4) Bioinks need to balance printability and biocompatibility, which are often conflicting. Specifically, high viscosity can increase shear forces during extrusion, potentially damaging cells, while low viscosity may lead to difficulties in maintaining basic structural integrity post-extrusion. Microgel-based bioinks may be a solution to the contradiction. The microgels loaded with cells act as the dispersed phase, forming the primary network through close packing. Simultaneously, the precursor of hydrogel infiltrates the pores between microgels as the continuous phase, establishing the secondary network. This dual-network structure harmonizes printability and biocompatibility.
(5) Loading growth factors is an important mean of functionalizing GelMA bioinks. However, concerns about the long-term instability and possible inactivation of growth factors have led to the need for improved encapsulation techniques and delivery systems for growth factors. Furthermore, the actual metabolism of encapsulated growth factors in the living microenvironment requires more detailed *in vivo* experiments.
(6) Bioprinting has now entered the era of 4D bioprinting. In 4D bioprinting technology, bioink is expected to respond to the external microenvironment or internal cells, performing one or even more functions over time. Specifically, the bioink can respond to stimuli such as water, temperature, light, pH, and magnetism to change the 4D bioprinting structure. However, most current bioinks are still only responsive to one stimulus, and the deformation of 4D bioprinted structures is limited to simple changes at the macroscopic scale. In contrast, the microenvironment in an organism is synergistically regulated in multiple ways, and cellular activity is affected by multiple stimuli, such as neuromodulation and humoral regulation. Therefore, the design of novel smart bioinks with multiple stimulus responses is the key to applying 4D bioprinting to bone tissue regeneration.
(7) The occurrence of combined defects is common in clinical practice, especially around bone joints. This multisystem defect necessitates a comprehensive approach, addressing not only bone tissue repair but also the reconstruction of cartilage, tendons, and muscle units. Therefore, research and treatment strategies for these multisystem defects are becoming increasingly important. The synchronous differentiation of cells from different lineages relies on bioinks with multi-directional regulatory capabilities, while the construction of composite tissues depends on the precise structure of 3D printing scaffolds.

In conclusion, although GelMA-based bioink is still a long way from clinical practice in bone tissue regeneration, more and more innovative studies are focused on developing GelMA functional bioink with a higher degree of matching between bone tissue defects, making it become one of the most promising bioink for bone tissue regeneration. We believe that therapeutic strategies based on cell-loaded GelMA scaffold *trans*-plantation will be implemented in the near future for treating bone tissue injuries.

## Ethics approval and consent to participate

This review manuscript does not involve animal experiments or clinical trials, so there is no Ethics approval and consent to participate.

## CRediT authorship contribution statement

**Yaru Zhu:** Writing – original draft, Validation, Methodology, Investigation. **Xingge Yu:** Writing – original draft, Validation, Methodology, Investigation. **Hao Liu:** Validation, Investigation. **Junjun Li:** Validation, Investigation. **Mazaher Gholipourmalekabadi:** Investigation, Validation. **Kaili Lin:** Writing – review & editing, Supervision, Project administration, Methodology, Funding acquisition, Conceptualization. **Changyong Yuan:** Writing – review & editing, Project administration, Methodology, Investigation, Conceptualization. **Penglai Wang:** Writing – review & editing, Supervision, Project administration, Methodology, Conceptualization.

## Declaration of competing interest

The authors declare that they have no known competing financial interests or personal relationships that could have appeared to influence the work reported in this paper.
